# Impact of Primary Health Care Reforms in Quebec’s Health Care System: A Systematic Review

**DOI:** 10.3390/healthcare14203373

**Published:** 2026-10-09

**Authors:** Jean-Denis Lalonde, Jean-Baptiste Gartner, Frédéric Bergeron, Elena Tànfani, Paolo Landa

**Affiliations:** 1 Department of Family Medicine, Faculty of Medicine and Health Sciences, McGill University, Montréal, QC H3G 2M1, Canada; jean-denis.lalonde@mail.mcgill.ca; 2 Département de Management, Faculté des Sciences de L’Administration, Université Laval, Québec City, QC G1V 0A6, Canada; jean-baptiste.gartner@fsa.ulaval.ca; 3 Axe Santé des Populations et Pratiques Optimales en Santé (SP-POS), Centre de Recherche du Centre Hospitalier Universitaire de Québec, Québec City, QC G1S 4L8, Canada; 4 Centre de Recherche de L’Institut Universitaire de Cardio-Pneumologie de Québec, Université Laval, Québec City, QC G1V 0A6, Canada; 5 VITAM, Centre de Recherche en Santé Durable, Université Laval, Québec City, QC G1V 0A6, Canada; 6 Centre de Recherche du CISSS de Chaudière-Appalaches, Lévis, QC G6V 3Z1, Canada; 7 Centre de Recherche en Gestion des Services de Santé (CRGSS), Université Laval, Québec City, QC G1V 0A6, Canada; 8 Bibliothèque-Direction des Services-Conseils, Université Laval, Québec City, QC G1V 0A6, Canada; frederic.bergeron@bibl.ulaval.ca; 9 Dipartimento di Economia, Università degli Studi di Genova, 16126 Genova, Italy; elena.tanfani@unige.it; 10 Département D’Opérations et Systèmes de Décision, Faculté des Sciences de L’Administration, Université Laval, Québec City, QC G1V 0A6, Canada; 11 Groupe de Recherche en Écologie Buccale (GREB), Université Laval, Québec City, QC G1V 0A6, Canada; 12 Centre Interuniversitaire de Recherche sur les Réseaux D’Entreprise, la Logistique et le Transport (CIRRELT), Québec City, QC G1V 0A6, Canada

**Keywords:** primary health care, Quebec, Canada, general practitioners, family medicine, health care reform, health services accessibility, health equity, systematic review

## Abstract

**Highlights:**

**What are the main findings?**

- Two decades of primary care reform in Quebec were accompanied by substantial organisational transformation: the expansion of Family Medicine Groups, the formalisation of patient attachment through centralised waiting lists, and the progressive integration of nurses and other professionals.
- Improvements in structural access (attachment rates, organisational mechanisms) were not consistently accompanied by improvements in timely access, patient-reported experience, health outcomes, or equity; the comparative evaluative evidence on patient-level effects is small (35 comparative evaluative reports, including one randomised trial) and mostly modest in magnitude.

**What are the implications of the main findings?**

- Organisational reforms alone are insufficient to address systemic constraints such as workforce distribution, physician remuneration models, and the imbalance between care demand and service capacity.
- Future policies should prioritise effective access, explicit equity-focused interventions targeting socially and medically vulnerable groups, and stronger evaluation frameworks based on patient-reported and longitudinal outcomes.
- This synthesis covers the reform cycle that closed in 2023 and therefore provides the baseline of evidence against which the subsequent phase of Quebec primary care policy can be assessed.

**Abstract:**

Introduction: Primary health care (PHC) reforms in Quebec between 2000 and 2023 aimed to improve accessibility, continuity, efficiency, and equity through organisational transformation, notably the Family Medicine Groups (FMGs), centralised waiting lists (Guichets d’accès), the advanced access model, and legislative instruments such as Bill 20. The evidence on their cumulative impact remains fragmented across disciplines, outcome domains, and study designs. Objective: To systematically identify, appraise, and synthesise the empirical evidence on the association between PHC reforms implemented in Quebec during the 2000–2023 reform cycle and organisational transformation, accessibility, health outcomes, costs, and equity, with mental health service organisation treated as a distinct policy stream outside the scope of this review. Methods: This systematic review followed the PRISMA 2020 statement and a protocol registered with PROSPERO (CRD42023421145). PubMed, EMBASE, Web of Science, and CINAHL were searched on 7 November 2023 and the searches were rerun on 3 September 2026, complemented by structured searches of Quebec governmental and institutional repositories and reference-list checking. Screening, extraction, and appraisal (Mixed Methods Appraisal Tool, MMAT 2018) were performed in duplicate. Reports were analysed for policy documents, implementation, descriptive and qualitative studies, and comparative evaluative studies, and synthesised narratively by reform mechanism and outcome domain, following SWiM guidance for quantitative evidence. Results: 192 reports were included (171 from the original search and 21 from the update), corresponding to 26 policy, institutional, review, or commentary documents, 131 implementation, descriptive, or qualitative studies, and 35 comparative evaluative studies, including one pragmatic randomised trial with reported results. Policy documents and implementation studies consistently describe organisational transformation and rising attachment rates. Comparative evaluative studies report that FMG enrolment and centralised-waiting-list attachment were associated with modest reductions in avoidable emergency department use and improvements in guideline-concordant care processes, whereas quasi-experimental evaluations of enrolment incentives found no meaningful population-level gain in affiliation. Evidence on health outcomes and costs is limited and largely proxy-based; socioeconomic and geographic gradients in access persisted across the period. Early evaluations of the Primary Care Access Points (2024–2026) report high patient-reported unmet needs when no physician appointment is obtained. Conclusions: The reforms were accompanied by substantial structural change and by limited, uneven, and mostly modest associations with patient-level access, outcomes, and equity. Confidence in these conclusions is moderate for organisational and attachment-related findings, low for health outcomes and costs, and constrained throughout by incomplete reporting and the scarcity of counterfactual designs.

## 1. Introduction

Primary health care (PHC) constitutes the foundation of equitable and efficient health systems. The World Health Organization and UNICEF define PHC as a whole-of-society approach designed to ensure the highest attainable standard of health and well-being through prevention, treatment, rehabilitation, and palliative care delivered as close as possible to people’s everyday environments [1]. Evidence accumulated over several decades indicates that strong primary care systems are associated with improved health outcomes, greater patient satisfaction, enhanced care coordination, and lower overall health expenditures [2]. In contrast, weak PHC structures are associated with avoidable hospital admissions, fragmented service delivery, overuse of emergency departments for non-urgent conditions, and persistent inequities in access to care [3,4,5].

In Canada’s decentralised but universal health system, provincial governments are responsible for the organisation and delivery of care within the framework of the Canada Health Act. Quebec, the second most populous province, undertook several major primary care reforms over the past two decades aimed at improving accessibility, continuity, equity, and efficiency of services [6,7]. The introduction of Groupes de médecine de famille (Family Medicine Groups, FMGs) in 2002–2003 and the adoption of Bill 20 in 2015 represent two of the most ambitious policy shifts in this trajectory, intended to expand access to general practitioners (GPs) and promote better use of medical resources through formal patient enrolment and the integration of multidisciplinary teams [8,9,10,11]. These reforms were complemented by parallel initiatives, including the gradual deployment of centralised waiting lists (Guichets d’accès) for patients without a family physician [12,13,14], the introduction of the advanced access scheduling model [15,16,17,18], the expansion of nurse practitioner roles [19,20,21,22,23], and reforms in physician remuneration mechanisms [24,25,26,27]. Because the institutional vocabulary of the Quebec system is unfamiliar to many international readers, Table 1 provides a glossary of the organisational entities, access mechanisms, and policy instruments referred to throughout the review.

Despite these initiatives, access to primary care in Quebec remains a persistent public concern. A substantial proportion of citizens still lack attachment to a family physician [28,29,30,31], experience long waiting times for appointments [32,33], and continue to rely on emergency departments for problems that could be addressed in primary care settings [34,35,36,37,38]. Regional disparities between urban, rural, and remote areas remain pronounced [39,40,41,42], and socioeconomic gradients in access and experience of care have proven remarkably resilient to structural reforms [14,43,44,45,46].

In addition, the COVID-19 pandemic exposed and intensified vulnerabilities in the organisation and delivery of primary care, putting unprecedented pressure on GPs, accelerating the deployment of telehealth, and revealing disparities between regions and population groups [47,48,49,50,51,52]. These developments have renewed policy interest in understanding whether structural reforms achieved their intended outcomes in terms of accessibility, health outcomes, cost containment, and equity. Numerous studies and governmental reports have evaluated specific interventions (FMGs, advanced access, centralised waiting lists, nurse practitioner integration, performance targets), but the evidence remains fragmented and heterogeneous, with limited synthesis across policy periods, outcome domains, and population subgroups [53,54,55,56,57].

The present systematic review therefore aims to identify, appraise, and synthesise the empirical evidence on the impact of PHC reforms in the Quebec public health care system over the 2000–2023 reform cycle. Its specific objectives are (i) to map the evidence base by reform mechanism, outcome domain, and study design; (ii) to distinguish, within that evidence base, policy implementation, implementation and descriptive evidence, and comparative evaluative evidence capable of estimating change associated with a reform; (iii) to synthesise the direction and consistency of findings on organisational transformation, accessibility, health outcomes, costs, and equity, with explicit attention to the distinction between formal (or structural) access and effective (or timely and relational) access, to the heterogeneity of implementation across regions and organisational settings, and to socially, medically, and geographically vulnerable populations; and (iv) to identify evidence gaps. The organisation of mental health services, which followed a distinct policy stream over the period, is outside the scope of this review and is the object of a separate systematic review by the same team (Section 2.2).

## 2. Materials and Methods

### 2.1. Design and Reporting

This study is a systematic review with narrative synthesis, aimed at synthesising the evidence on the impact of PHC reforms in the Quebec public health care system between 2000 and 2023. It was conducted following the Preferred Reporting Items for Systematic Reviews and Meta-Analyses (PRISMA) 2020 statement [58] and the PRISMA-S extension for the reporting of the literature searches [59], based on a predefined protocol published in advance [60] and prospectively registered with PROSPERO (CRD42023421145). The completed PRISMA 2020 checklist is provided as Supplementary Material S1. Given the heterogeneity of study designs, outcome measures, and conceptual frameworks, a narrative synthesis approach was adopted, structured according to the Synthesis Without Meta-analysis (SWiM) reporting guideline for the quantitative evidence [61] and the guidance of Popay et al. for the integration of qualitative and mixed-methods evidence [62].

### 2.2. Eligibility Criteria

Eligibility was defined using a PICOTS (Population, Intervention, Comparator, Outcome, Timing, Setting) framework aligned with the protocol [60]. The population was the adult population of Quebec and the professionals and organisations delivering primary care to it. The intervention was any PHC reform, policy instrument, or organisational model introduced or expanded in Quebec during the 2000–2023 reform cycle, operationalised as one of the following reform mechanisms: (a) FMG implementation and team-based practice (including nurse and nurse practitioner integration); (b) centralised waiting lists and patient attachment mechanisms (GACO, GAMF, and, in the search update, the GAP); (c) the advanced access scheduling model; (d) legislative and accountability instruments (Bill 20, performance targets, PREM/AMP workforce regulation); (e) physician remuneration and incentive reforms; (f) digital access tools (e-booking, eConsult, electronic records, telehealth); and (g) targeted access or integration programmes deployed within primary care (case management, navigation, chronic disease management programmes). Comparators, where present, were pre-reform periods, non-exposed populations or organisations, or alternative organisational models. Outcomes were organisational transformation, accessibility (structural, timely, and relational), health outcomes and care processes, costs and resource use, and equity. Timing was January 2000 onwards; the setting was primary care in Quebec, alone or as one jurisdiction in a multi-jurisdictional study reporting Quebec-specific results.

In response to the concern that the protocol formulation “activities involving general practitioners or family physicians” could admit reports with no defined reform as exposure, each included report was, during data extraction, explicitly linked to one or more of the reform mechanisms (a)–(g) above. Reports that described the organisation or performance of Quebec primary care without evaluating a named reform mechanism (for example, disease-specific practice-pattern surveys or referral-pattern studies) were retained only when they provided outcome data for a period and organisational setting relevant to the reforms and were classified as contextual descriptive evidence (Stream B, Section 2.7) rather than as reform-evaluative evidence. This distinction between contextual and reform-evaluative reports was applied by two reviewers (PL, JDL), with disagreements resolved by a third (JBG).

Eligible designs were systematic reviews, randomised controlled trials, cohort studies, case–control studies, cross-sectional studies, quasi-experimental designs, natural experiments, interrupted time series, qualitative studies, mixed-methods research, and governmental or institutional evaluative reports. Systematic reviews were retained as contextual sources and their included primary studies were not counted a second time when they were already in the corpus (Section 2.7). The grey literature was eligible when it reported empirical data, including reports of the Commissaire à la santé et au bien-être [63,64,65,66,67], the Conseil du statut de la femme [68], the Institut national de santé publique du Québec (INSPQ), the Institut de la statistique du Québec [69], the Ministère de la Santé et des Services sociaux [70], and the Fédération des médecins omnipraticiens du Québec [71].

Reports were excluded if they were systematic review protocols, editorials, letters, or news items without empirical content; conference abstracts; did not focus on Quebec; did not evaluate or describe a reform mechanism or its context; or were conducted exclusively in paediatric or adolescent populations, or in mixed samples of adults and minors that did not report results for adults separately. One report on an integrated youth service for people aged 12–25 years [72], which did not report results for adults separately, was retained as an explicit exception to this rule, because its population partly overlaps the adult population (18–25 years) and it documents the role of family physicians in a reform-era service model; it is used as contextual evidence only (Section 3.4) and does not contribute to the findings. Commentaries and editorials were retained as contextual documents (Stream A, Section 2.7) when they discussed a Quebec primary care reform instrument; other commentary-style publications were excluded. Published protocols of primary studies were retained when they described an intervention within scope that was being implemented in Quebec primary care, because they document work in progress that could yield relevant evidence; they are used as implementation evidence only and contribute no effect estimates [73,74]. Only reports published in English or French from January 2000 onwards were considered.

**Figure 1:**
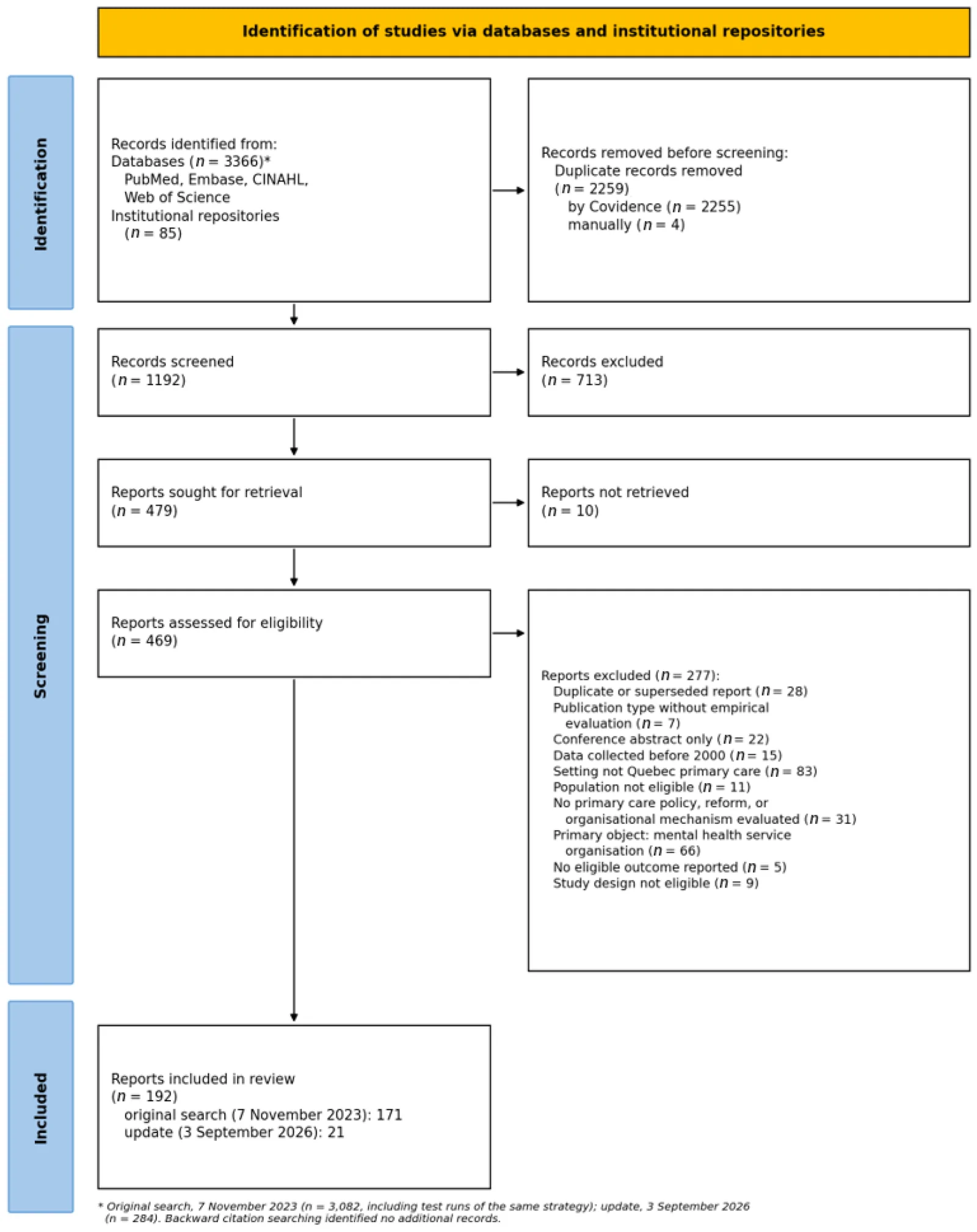
PRISMA 2020 flow diagram of study selection, including the 2026 search update.

One exclusion was introduced after protocol registration and is reported here in full, in accordance with PRISMA item 24c. Studies whose primary object was the organisation, funding, or delivery of mental health services were excluded at full-text screening. The decision was taken by the two screening reviewers (PL, JDL) in consultation with the third reviewer (JBG) at the outset of full-text screening, on the basis of the titles and abstracts retained at the previous stage and before any data extraction or synthesis had been undertaken; it was therefore made without knowledge of the findings of the reports concerned. The rationale is conceptual: mental health care in Quebec over the period was governed by a distinct sequence of policy instruments (the Plans d’action en santé mentale 2005–2010 and 2015–2020, and the Programme québécois pour les troubles mentaux from 2020), with their own mechanisms (shared care, psychiatric consultation–liaison, access to psychological services), targets, and funding, whose trajectory was only partly determined by the attachment-based reforms under study. Synthesising the two streams as a single object would have attributed to the FMG, waiting-list, and Bill 20 reforms effects that belong to a separate policy sequence. “Primary object” was operationalised as follows: a report was excluded when the intervention evaluated, or the outcome measured, was a mental health service or programme (for example, collaborative or shared mental health care, integration of psychologists, access to psychotherapy); it was retained when mental health appeared as one clinical area among others within a general primary care intervention, as a comorbidity in a population defined otherwise (frequent emergency users, multimorbid or socially vulnerable patients), or as a determinant of access. This rule accounts for 66 of the 277 full-text exclusions (Figure 1); the excluded reports are identified in Supplementary Material S4 (primary reason 8), and the implications of the restriction for the evidence map are examined in Section 3.8.4 and discussed in Section 4.1 and Section 4.5. The mental health stream is the object of a separate systematic review being conducted by the same team.

### 2.3. Information Sources and Search Strategy

Four electronic databases were searched: PubMed, EMBASE (Ovid), Web of Science Core Collection, and CINAHL (EBSCO). The strategies were developed by an academic health sciences librarian (FB) and combined controlled vocabulary (e.g., MeSH and Emtree terms) with free-text keywords related to primary care, general practitioners and family physicians, health care reform, health services accessibility, equity, and Quebec, limited to publications from January 2000 onwards. Test runs of the same strategy, used to estimate the screening workload, were imported into Covidence and are counted with the original search; the four database searches were run in full on 7 November 2023, when screening began, and rerun with identical syntax on 3 September 2026, limited in the update to records added since the original search. The full database-specific strategies, with platform, coverage, syntax, limits, dates, and number of records retrieved at each run, are reported in Supplementary Material S2 in accordance with PRISMA-S [59].

The grey literature was retrieved through structured browsing of the online repositories of the following institutions, at both search dates: the Commissaire à la santé et au bien-être, the INSPQ, the Institut de la statistique du Québec, the Ministère de la Santé et des Services sociaux (publications catalogue), the Institut national d’excellence en santé et en services sociaux, the Fédération des médecins omnipraticiens du Québec, the Conseil du statut de la femme, and the Santécom catalogue of the Quebec health and social services network libraries. For each source, Supplementary Material S2 reports the updated search strategy. Backward citation searching of the reference lists of all included reports was also performed at both dates. Records retrieved from the institutional repositories were imported into Covidence in a single batch and screened with the same procedure as, and together with, the database records; they are therefore reported in the identification box of a single-arm PRISMA flow diagram (Figure 1). Backward citation searching identified no report that had not already been retrieved by the database or repository searches.

### 2.4. Selection Process

All records were imported into Covidence [71], and duplicates were removed automatically and by manual inspection. Two reviewers (PL, JDL) independently screened titles and abstracts and then assessed full texts against the eligibility criteria; disagreements were resolved by discussion or by a third reviewer (JBG). Reports whose full text could not be located or obtained after requests to the authors and institutional libraries (*n* = 10) were recorded as “not retrieved” and were not assessed for eligibility. Each report excluded at full text was assigned a single primary exclusion reason from a taxonomy derived from the eligibility criteria of Section 2.2 and applied in a fixed hierarchical order, the recorded reason being the first criterion not met: (1) duplicate or superseded report (second record, translated version, summary, or interim report of a report already assessed); (2) publication type without empirical evaluation (editorial, commentary, systematic review protocol, evaluation design, questionnaire, or project entry without results); (3) conference abstract only; (4) data collected before 2000; (5) setting not Quebec primary care (study conducted outside Quebec or Quebec data not separable; hospital, specialist, integrated-care, or exclusively private setting); (6) population not eligible (paediatric or adolescent populations, or mixed samples without separate adult results); (7) no primary care policy, reform, or organisational mechanism (a)–(g) evaluated; (8) primary object mental health service organisation (post-protocol criterion, Section 2.2); (9) no eligible outcome reported; (10) study design not eligible. All full-text decisions were re-audited for this revision by two reviewers (PL, JDL): the labels of the original Covidence project, four of which were inherited from a screening-software template, were retired and every excluded report was re-coded to the taxonomy above; the eligibility of every included report was also re-checked against the criteria of Section 2.2, and five reports included in the submitted version were excluded at this re-audit (Section 3.1). The citation-level list of every report excluded at full text, with its primary reason and a one-line justification, and the crosswalk from the original to the re-audited labels, are provided in Supplementary Material S4.

### 2.5. Data Extraction

Data were extracted independently by two reviewers (PL, JDL, with ET for the update) into a predefined form piloted on ten reports and refined for consistency. The following information was extracted from each report: bibliographic details; project or programme to which the report belongs; study design; setting, population, and sample size; data-collection years; reform mechanism(s) evaluated, coded to categories (a)–(g) of Section 2.2; comparator and follow-up where applicable; outcome definitions and, for quantitative reports, the principal numerical results with their measures of uncertainty; definition of vulnerability where applicable; role and activities of GPs; organisational context; and the authors’ interpretation.

In accordance with PRISMA 2020, a distinction is drawn between reports (individual publications) and studies (the underlying investigations). Several publications in this field arise from the same research programme or dataset (for example, the Montreal and Montérégie population surveys of 2005 and 2010, the FMG administrative-data evaluations, the multimorbidity trial and its process evaluation [75,76]). Each report was therefore assigned a study or programme identifier; the report-to-study/programme crosswalk is provided in Supplementary Material S5, and the consistency of findings was assessed across independent studies or programmes rather than across reports.

### 2.6. Methodological Quality Appraisal

The methodological quality of the empirical primary studies was appraised with the Mixed Methods Appraisal Tool (MMAT), version 2018 [77], by two reviewers independently, with disagreements resolved by discussion or by a third reviewer. Only empirical primary studies can be appraised with the MMAT; systematic reviews, policy and institutional reports, and commentary-style publications were not appraised and were treated as contextual sources rather than as outcome evidence of equivalent standing (Section 2.7). Mixed-methods reports were appraised on the five mixed-methods criteria and, as required by criterion 5.5, on the criteria of their qualitative component and of the relevant quantitative component; the mixed-methods randomised trial [75] was thus appraised on the qualitative, randomised, and mixed-methods criteria. In keeping with MMAT 2018 guidance, no overall numerical score was computed; criterion-level judgements (“yes”, “no”, “can’t tell”) with a brief justification for each “no” and “can’t tell” are reported for every appraised report in Supplementary Material S3, and the distribution of judgements is summarised by category in Table 2. The appraisal was used as an analytical filter in the synthesis (Section 2.7), not as an exclusion criterion.

### 2.7. Synthesis Methods

Because the interventions, populations, designs, and outcome measures were heterogeneous, a meta-analysis was not undertaken; the assessment of poolability was made within outcome groups (Section 3.8) rather than on the basis of overall heterogeneity. The synthesis proceeded in four steps.

First, each report was assigned to one of three evidence streams according to the type of inference it can support. Stream A comprises policy, legislative, institutional, review, and commentary documents, which establish the reform timeline, intended mechanisms, and system-level indicators. Stream B comprises implementation, descriptive, and qualitative studies (cross-sectional surveys, uncontrolled administrative audits, qualitative and mixed-methods implementation studies), which describe organisational change, feasibility, professional roles, local adaptation, barriers, and patient or provider experience. Stream C comprises comparative evaluative studies (controlled observational studies with adjustment, difference-in-differences, interrupted time series, propensity-matched cohorts, and randomised trials), which can estimate change in access, utilisation, processes, or outcomes associated with a reform. Assignment was performed independently by two reviewers (PL, JDL) and is reported for each report in Supplementary Material S5.

Second, findings were coded to the reform mechanisms (a)–(g) and to the five outcome domains of the protocol (organisational transformation, accessibility, health outcomes and care processes, costs and resource use, equity), with cross-cutting codes for vulnerability, implementation heterogeneity, and the distinction between structural and effective access. For each quantitative finding, the direction of effect was coded (favourable, unfavourable, null, or mixed) relative to the reform’s intended objective, based on the point estimate and its confidence interval where reported; qualitative findings were coded as supporting, qualifying, or contradicting the intended mechanism. Contradictory findings within a report were coded separately by outcome. Coding was performed by one reviewer (PL) and verified by a second (JDL); discrepancies were resolved by consensus.

Third, findings were synthesised by reform mechanism and outcome domain, giving priority within each cell to Stream C evidence and using Stream B and Stream A evidence to interpret mechanisms, implementation conditions, and context. The MMAT appraisal informed this step: findings resting on reports with a high proportion of “can’t tell” judgements on the criteria most relevant to the outcome (for example, confounding and intervention fidelity for non-randomised studies, non-response bias for descriptive studies) were treated as lower-confidence, and this is stated explicitly in the Results. Economic evidence was distinguished from resource-utilisation proxies, and equity evidence was defined as explicit stratified or distributional analyses rather than the mention of vulnerable populations.

Fourth, the synthesis was consolidated in a Summary of Findings matrix reporting, for each reform mechanism and outcome domain, the number of reports and independent studies, the predominant designs, the direction and consistency of findings, and a qualitative confidence statement (high, moderate, low, very low) based on the study designs available, the MMAT judgements on the criteria most relevant to the outcome, the consistency of direction across independent studies, and the directness of the outcome measure. A formal GRADE or GRADE-CERQual assessment was not undertaken because the evidence in most cells is neither a set of comparable effect estimates nor a set of qualitative findings on a single phenomenon; the confidence statements are therefore reviewer judgements applying the same four considerations, and their rationale is given in Supplementary Material S6.

Patients and the public were not involved in the design or conduct of this study.

### 2.8. Temporal Boundaries and Amendments to the Protocol

Two temporal boundaries apply and rest on different justifications. The first concerns the policy period under study: the review covers the reform cycle that began with the creation of the FMGs in 2002–2003 and closed with the consolidation phase following Bill 20, together with the centralised waiting lists and the advanced access model. This cycle shares a common policy logic: individual patient attachment to a family physician organised through multidisciplinary group practices and can therefore be synthesised as a coherent object. The GAP, introduced in 2022, marks the transition toward a phase built on collective access management; reports evaluating the GAP were eligible in the update because they evaluate the closing phase of the cycle and the transition from it, and they are presented separately (Section 3.3.4). The second boundary concerns the currency of the evidence: eligibility was restricted to evidence evaluating the 2000–2023 reform cycle, but the search was not closed in 2023; it was rerun in September 2026 so that reports published in 2024–2026 evaluating reforms within scope are represented.

Four amendments to the published protocol [60] are reported in accordance with PRISMA item 24c. First, the protocol specified a search window ending in January 2022; the searches were run in November 2023 and updated in September 2026. Second, reports whose primary object was mental health service organisations were excluded at full-text screening, as described in Section 2.2. Third, the composition of the review team changed during the conduct of the study, and data extraction was performed by the authors listed in the contributions statement rather than by the four reviewers named in the protocol. Fourth, in response to peer review, the synthesis was restructured into evidence streams with a Summary of Findings matrix and the unit of counting was clarified as the report with a crosswalk to studies; no changes were made to the outcome domains or to the appraisal instrument.

## 3. Results

### 3.1. Study Selection and Composition of the Evidence Base

The study selection process is presented in Figure 1, which uses the PRISMA 2020 template and reports the two search runs (7 November 2023 and 3 September 2026) cumulatively. The database searches identified 3366 records: 3082 from the original search of 7 November 2023, including the test runs of the same strategy, and 284 from the update of 3 September 2026. The institutional repositories yielded 85 records, of which 74 were unique after deduplication and were screened together with the database records; 25 of the included reports came from this source. After the removal of 2259 duplicates (2255 identified by Covidence and 4 manually), 1192 records were screened on title and abstract, of which 713 were excluded; 479 reports were sought for retrieval, 10 of which could not be retrieved, and 469 reports were assessed for eligibility. Of these, 277 were excluded, with a single primary reason drawn from the taxonomy of Section 2.4: setting not Quebec primary care (*n* = 83), primary object mental health service organisation (*n* = 66), no primary care policy, reform, or organisational mechanism evaluated (*n* = 31), duplicate or superseded report (*n* = 28), conference abstract only (*n* = 22), data collected before 2000 (*n* = 15), population not eligible (*n* = 11), study design not eligible (*n* = 9), publication type without empirical evaluation (*n* = 7), and no eligible outcome reported (*n* = 5); the citation-level list, with the stage at which each decision was taken, and the crosswalk from the labels originally recorded in Covidence are given in Supplementary Material S4. These figures incorporate the re-audit of the included reports conducted for this revision, which led to six changes relative to the submitted version: one report was identified as a duplicate citation of a report already included [79] and was merged, and five reports were excluded because they did not meet the eligibility criteria of Section 2.2 (two documents without empirical results, namely a survey questionnaire and an evaluation design and baseline document; one conference-supplement abstract; and two reports whose population was perinatal, paediatric, or adolescent). In addition, one commentary excluded before the first submission was reinstated under the rule on commentaries of Section 2.2 [80]. In total, 192 reports were included in the synthesis, 171 from the original search and 21 from the 2026 update [72,81,82,83,84,85,86,87,88,89,90,91,92,93,94,95,96,97,98,99,100].

The composition of the evidence base is summarised in Table 3. By evidence stream, 26 reports are policy, legislative, institutional, review, or commentary documents (Stream A), 131 are implementation, descriptive, or qualitative studies (Stream B), and 35 are comparative evaluative studies (Stream C), comprising 34 longitudinal, quasi-experimental, or controlled observational reports and one pragmatic mixed-methods randomised trial [75], the only randomised trial in the corpus that reports results. The dominance of Stream B, which describes the reforms and their implementation but cannot estimate change attributable to them, is the single most important feature of this literature and conditions the confidence that can be placed in each finding below. Research activity increased over time and after each major reform, and the 21 reports added by the update are concentrated on the closing phase of the cycle: nine evaluate attachment and access mechanisms (centralised waiting lists, enrolment policies, the GAP), four evaluate advanced access or clinic-level access practices, three evaluate interprofessional collaboration or nursing roles, two evaluate eConsult, and three evaluate targeted programmes (chronic pain, low back pain, peer support).

Across the literature, reforms are consistently framed as responses to structural pressures, including chronic physician shortages [29,30,41,101], increasing demand from an ageing and multimorbid population [102,103,104], fragmentation of services [9,105,106], and persistent accessibility challenges [28,31,32,33]. The evidence does not converge toward a single assessment of effectiveness; rather, it documents multiple layers of partial success and persistent systemic constraints, with substantial variation in implementation fidelity, organisational maturity, and population reach. The sections that follow present the synthesis by outcome domain, identifying in each case which findings rest on comparative evaluative evidence and which on descriptive or qualitative evidence.

### 3.2. Primary Care Reforms and Organisational Models

Policy documents and institutional evaluations describe the FMG model as the cornerstone of the reform cycle [6,7,9,107,108,109] and document its diffusion: from 19 accredited groups in 2003 to more than 350 by the late 2010s, covering mostly family physicians. These documents establish that structural change occurred; they do not, by themselves, establish that the change affected performance.

Implementation and descriptive evidence (Stream B). The implementation studies consistently report that FMG accreditation was accompanied by the formalisation of patient enrolment and the gradual integration of multidisciplinary teams, including nurses, social workers, pharmacists, and other allied professionals [75,110,111,112,113,114,115,116]. The organisational diversity of Quebec primary care has been extensively documented: beyond FMGs, the literature describes CLSCs, private group clinics, solo practices, network clinics, and university family medicine units [107,117,118,119,120,121]. Cross-sectional comparisons of these organisational forms, most of them drawn from the 2005 and 2010 Montreal–Montérégie population surveys, report that integrated and team-based models score higher than solo and traditional private clinics on continuity, coordination, comprehensiveness, and provision of clinical preventive services [79,106,122,123,124], whereas solo clinics were reported to privilege their established patient panel and to offer limited preventive and urgent-care capacity [120]. These comparisons are adjusted for patient characteristics but not for physician self-selection into organisational models, and the MMAT judgements on confounding were “can’t tell” for most of them; they establish associations between organisational form and reported care processes, not the effect of moving a practice from one form to another.

A more critical examination shows that organisational change was often more formal than functional. Qualitative and mixed-methods studies report that, despite the introduction of team-based structures, clinical practice remained largely physician-centred, with limited redistribution of tasks [14,50,125,126,127], and that communication and interprofessional collaboration were frequently suboptimal even within ostensibly integrated FMGs [112,115,125]. Professional norms, hierarchical relationships, regulatory constraints, and entrenched billing structures are identified as limiting the depth of transformation. The 2026 update adds convergent evidence from the closing phase: a mixed-methods pre–post implementation project in a Montreal FMG found that, even after targeted training, family physicians still provided over 80% of consultations and nearly half of the professional appointments reserved for unattached patients remained unfilled, while nursing, social-work, and physiotherapy capacity was vacant for a substantial share of the intervention period [96]; and a qualitative multiple-case study of case-management scale-up identified fee-for-service remuneration as a direct barrier to physician participation in interprofessional service plans [88]. A recent expert-committee analysis estimated that physician-based models serve approximately 900 patients per full-time-equivalent physician against roughly 2000 for interdisciplinary teams operating at a 3:1 professional-to-physician ratio but noted that this capacity has been realised only unevenly [100].

Implementation heterogeneity is a recurring theme. Studies consistently report significant variation across regions and organisations in resources, governance, team composition, and operational capacity [15,18,54,116,128]. Some FMGs operate as highly integrated teams with extensive nurse involvement and structured chronic disease management protocols, while others function as loosely coordinated networks [16,76,113]. A ten-year retrospective portrait of the advanced access model found that diffusion progressed, but full implementation remained uneven and partial, with substantial differences between early and late adopters [18]. Two update reports characterise this heterogeneity more precisely: a latent-class analysis of 335 providers in university-affiliated clinics identified six distinct advanced access practice profiles, with self-reported third-next-available appointments ranging from a median of 4 days in a “rural agility” profile to about 9 days in urban delegating and comprehensive profiles [82]; and a survey of 44 of 46 university-affiliated FMGs found that no residency programme was compatible with all four pillars of advanced access and that 82% kept less than 20% of the schedule open for urgent needs [84]. A realist evaluation of four quality-improvement initiatives, including one advanced access implementation, identified clinical champions as a mechanism through which local adoption is sustained, and their absence as a reason for stalled implementation [99]. Comparative analyses with Ontario’s Family Health Teams suggest that Quebec’s model was less ambitious in team composition and population accountability [7,55,56].

Policy instruments such as Bill 20 introduced performance targets, minimum patient panel requirements, and accountability mechanisms [8,10,11]. The literature on their effects is almost entirely descriptive or qualitative: it reports concerns about increased administrative burden, changes in physician behaviour prioritising measurable outputs, and risks of adverse selection of patients with simpler profiles [9,26,50,129], and it documents that fee-for-service incentives may not align with the goals of team-based reform and that the structure of compensation shapes both the volume and the composition of services delivered [24,25,27]. Solo clinics were reported to be particularly resistant to organisational change, with limited involvement in preventive activities and after-hours coverage [120,130].

Comparative evaluative evidence (Stream C). The integration of nurses and nurse practitioners (NPs) is the organisational dimension for which comparative evidence is most developed, although it remains thin. Descriptive and qualitative work documents the expansion of nursing roles in FMGs and reports favourable associations with chronic disease management, patient experience, and care coordination [22,111,112,113,114,116,131], identifies institutional support, role clarity, and willingness to redistribute clinical responsibilities as conditions of successful NP integration [19,20,21,23,126,127,132,133], and, in the update, describes how a patients-as-trainers intervention was perceived to change registered nurses’ patient-engagement practices in primary care clinics [97]; regulatory and professional barriers continue to constrain NP deployment, particularly in rural and underserved areas [23,127,132]. The only comparative report is a prospective observational study in six long-term care facilities, which found that NP introduction was associated with measurable operational and cost benefits [133]. No controlled evaluation of nurse-led or NP-led primary care clinics on utilisation outcomes met the eligibility criteria; a longitudinal matched evaluation of NP-led clinics identified in the update was available only as a conference abstract and was therefore excluded (Section 2.2).

### 3.3. Accessibility of Primary Care

Accessibility is the most extensively analysed domain and the primary policy objective of the reforms. The synthesis distinguishes structural access (attachment, panel size, existence of organisational mechanisms), effective or timely access (obtaining care when needed), and relational continuity.

#### 3.3.1. Structural Access and Attachment

Streams A and B. Institutional reports and cross-sectional studies agree that attachment rates rose over the period and that the centralised waiting lists (GACOs, later GAMF) were the mechanism through which several hundred thousand previously unattached patients were attached to a family physician between 2008 and 2018, with acceleration after Bill 20 [12,13,14,37,45,79,117,128,129,134,135,136]. The waiting-list evaluations are largely descriptive analyses of registry data and provider surveys; they document throughput and the characteristics of attached patients but do not compare attachment with a counterfactual.

Stream C. Two comparative reports in the update qualify the structural picture. A quasi-experimental evaluation using difference-in-differences and interrupted time-series designs on population-level administrative data compared Quebec with British Columbia and found that neither the 2003 enrolment policy for vulnerable patients nor the 2009 general enrolment policy produced a meaningful population-level improvement in patient–physician affiliation, measured as usual-provider continuity and an administrative proxy for reporting a regular doctor: difference-in-difference estimates for the 2003 policy ranged from −0.25 to −0.95 percentage points among regular users, and slope and level changes for the 2009 policy were small and inconsistent in sign [89]. The authors attribute the null result in part to the pre-existing scarcity of supply, which made free choice of a family physician largely theoretical. Conversely, a population-based retrospective cohort study of patients attached between 2008 and 2019 found that, after adjustment, patients attached through the centralised waiting list had a 4.7% lower rate of non-urgent emergency department visits in the year following attachment than patients attached through other routes (incidence rate ratio 0.952; 95% CI 0.946–0.960) [81]. Read together, these two reports indicate that enrolment incentives did not by themselves increase affiliation at the population level, whereas the waiting-list route, once attachment occurred, was associated with a small reduction in non-urgent emergency use. Both reports rest on administrative data with adjustment for observed characteristics; MMAT judgements on residual confounding were “can’t tell” and the estimates should be read as adjusted associations. An earlier longitudinal cohort found that attachment through the waiting list was followed by improved access and continuity as reported by patients [136].

#### 3.3.2. Effective and Timely Access

Despite structural gains, a consistent finding across streams is the persistence of limited effective access. Population surveys, in particular the Enquête québécoise sur l’expérience de soins and the Montreal–Montérégie surveys, repeatedly documented that, even among attached patients, obtaining a timely appointment for a same-day or next-day non-urgent need remained difficult, and that a sizeable share of the population resorted to walk-in clinics or emergency departments [3,31,32,33,35,64,66,137]. These are Stream B descriptive data; their strength is population coverage, their limitation is the absence of a counterfactual and, for several, an unassessable risk of non-response bias. A qualitative study in the update, drawing on interviews with unattached patients in Quebec and three other provinces, describes the concrete consequences of non-attachment: reliance on walk-in clinics, emergency departments, and private-pay providers, and difficulty obtaining specialist referrals, diagnostic requisitions, prescriptions, and approvals for public support programmes [92].

A key conceptual contribution of the Quebec literature is the distinction between structural and effective access [32,119,138,139]. Cross-sectional studies report that organisational characteristics such as panel size, nurse presence, after-hours arrangements, and advanced access scheduling are more strongly associated with patient-reported accessibility than attachment status itself [17,107,138,139]. The update adds the largest such analysis: a multilevel study of 104 multidisciplinary clinics with patient-reported experience data found that clinic-level practices (estimating demand and supply, opening schedules two to four weeks ahead, optimising consultations) were associated with lower odds of difficulty obtaining an appointment and of perceived unmet needs, whereas most individual patient characteristics, including social vulnerability, were not [95]. The literature also emphasises that formal attachment does not ensure relational continuity [31,140,141].

Organisational innovations such as advanced access are described as contributing to appointment availability [15,16,17,18,110,142], and digital tools have been deployed with the same aim [52,143,144,145]. For advanced access, however, the evidence remains descriptive: no controlled comparison of patient-reported timely access before and after its implementation was identified, and the update reports [82,84,99] document heterogeneity and partial implementation rather than effects.

#### 3.3.3. Emergency Department Use as a Barometer

The emergency department remains a recurring barometer of primary care access. Descriptive studies document its use by patients who could have been managed in primary care, particularly among diabetic patients, older adults, and those with chronic conditions [3,5,34,35,36,37,38,146]. The Stream C evidence is the most developed of the review. Quasi-experimental analyses of FMG enrolment reported modest reductions in avoidable emergency visits for diabetic patients [37,38]; a marginal-structural-model analysis of team-based care reported small effects on service utilisation [110,147]; and the update adds a 4.7% adjusted reduction in non-urgent emergency use after waiting-list attachment [81]. The magnitude of the associations is consistently modest, and their variation across patient subgroups, regional contexts, and stages of reform deployment [38,146,148,149] is as informative as their average.

#### 3.3.4. The Primary Care Access Points (GAP): Early Evaluations, 2024–2026

The GAP, introduced in 2022 to orient unattached patients while they await attachment, marks the transition from the attachment-based cycle to a collective access model. Four reports in the update provide its first evaluations; all are descriptive (Stream B) and were not available at the original search. A longitudinal multi-case qualitative study describes the organisational innovations developed around the GAP in three local territories (temporary nurse-led chronic-disease clinics, mobile proximity clinics, community-pharmacist trajectories, decentralised GAP nursing care), reporting that 40–70% of decentralised requests were completed without further referral and that nearly 20% of mobile-clinic cases were resolved without a physician appointment, while noting that the waiting-list requirement of a health-insurance card and postal address created barriers for refugees, recent immigrants, and children in protective care [85]. A retrospective cross-sectional analysis of 13,291 GAP requests found that 64% resulted in a face-to-face physician appointment, with higher adjusted odds for younger adults and for acute, medication-renewal, and administrative reasons, and marked territorial variation (no appointment available in 0.8% versus 11.4% of cases across two access points) [98]. A cross-sectional survey of 20,282 GAP users found that patient-reported unmet needs ranged from 17.9% after a family-physician appointment to 71.8% after receiving information or advice only, with younger patients, those in fair or poor health, and prior emergency users more likely to report unmet needs [86]. Finally, the eConsult service, scaled up in Quebec from 2021 as an asynchronous channel between primary care providers and specialists, was reported in provider surveys to have avoided a potentially unnecessary in-person specialist visit in more than 40% of cases [94], and a multi-jurisdictional case study documents the enabling factors of its scale-up [87]. These reports describe what the GAP delivers and to whom; they do not yet estimate its effect on attachment, emergency use, or outcomes, and the high unmet needs reported when no physician appointment is obtained indicate that reorientation to other services has not, so far, been experienced by patients as equivalent to physician access.

#### 3.3.5. Geographic and Complementary Dimensions

Rural and remote regions continued to experience provider shortages that limited the reach of reforms [39,40,41,42,50,69]. Spatial accessibility analyses reveal substantial intra-urban variation within Montreal, with deprived neighbourhoods facing higher travel times and lower provider densities [39], and some studies suggest that reforms benefited urban populations, where implementation conditions were more favourable [29,40,48]. Decentralised training programmes were associated with improved physician retention in one region, with uneven results [41,101]. Several adjacent interventions were evaluated for their contribution to access: national e-booking showed uneven uptake with risks of widening the digital divide [52,143,144]; telephone outreach by volunteer navigators was associated with improved access for patients from deprived neighbourhoods in a theory-based evaluation [150]; pharmacist-led initiatives were proposed to compensate for limited GP availability [151]; and the IMPACT cross-sector programme produced encouraging evidence on intersectoral navigation for vulnerable populations [152].

### 3.4. Health Outcomes and Care Processes

The impact of reforms on health outcomes is the most uncertain domain, and the distinction between care processes, utilisation proxies, and health outcomes proper is essential to its reading.

Care processes (Stream C). The strongest evidence concerns guideline-concordant processes. Longitudinal administrative-data analyses reported that FMG enrolment was associated with measurable improvements in recommended screening and chronic disease care processes [110,142,153], and that team-based models were associated with timelier physician follow-up after hospital discharge [148,149]. These analyses use enrolment as exposure with adjustment or inverse-probability weighting; their principal limitation, flagged by “can’t tell” MMAT judgements on intervention fidelity, is that enrolment is a formal status whose clinical content varied across FMGs.

Utilisation proxies (Stream C). Reductions in avoidable emergency visits and outpatient utilisation reported in some studies [5,34,36,37,38,81] are modest, context-dependent, and concentrated in specific subgroups (Section 3.3.3). In most cases, changes in utilisation did not translate into measurable changes in population health indicators such as mortality, morbidity, or quality of life [146,154].

Health outcomes. The only randomised trial results in the corpus come from a pragmatic mixed-methods randomised trial of patient-centred interdisciplinary care for multimorbidity, which demonstrated feasibility and acceptability with mixed results on clinical endpoints [75], and whose process evaluation [76] documents the implementation conditions; the two reports are counted as one study. Two further reports are published protocols of randomised evaluations of chronic disease management and of case management in FMGs, retained as evidence of work in progress (Section 2.2) and without trial results [73,74]. In the update, an observational repeated-measures evaluation of an integrated interdisciplinary primary care programme for chronic low back pain in four regions reported clinically meaningful improvement in pain interference for 69% of participants at six months, with larger proportions among those at high risk of chronicity, but without a comparison group [83]. Realist syntheses and case studies of chronic disease prevention and management programmes in FMGs identify enabling factors, including dedicated nursing time, structured protocols, self-management support, and case management for high-need users, together with persistent difficulties in scaling these interventions across heterogeneous settings [73,74,88,155,156,157,158,159,160]. In the evaluation of an integrated cardiometabolic risk prevention and management network in Montreal (1689 patients, about 40% of whom withdrew during the first year), the proportion of patients reaching clinical targets increased at 12 months, and closer coordination between the interdisciplinary teams and primary care physicians was associated with greater programme participation and better clinical results, in the absence of a comparison group [161]. Case management for frequent users with complex needs was associated with favourable patient and family experience, particularly for people with low health literacy, mental health comorbidity, or social vulnerability [74,156,157,162], and a qualitative single-case study of peer-support integration in a Montreal clinic serving a disadvantaged community reported perceived benefits in trust, navigation, and the resolution of therapeutic impasses [91]. COPD care trajectories analysed by sequence analysis reveal substantial heterogeneity with socioeconomic correlates [163,164].

A major limitation of this domain is the reliance on proxy indicators rather than direct health measures, which limits the assessment of effectiveness over the medium and long term [50,53,105], together with the difficulty of attributing outcomes to primary care reforms when demographic trends, social determinants, and other system changes operate simultaneously [46,50,165,166]. Validated instruments such as the Patient Experience of Integrated Care Scale offer more refined patient-centred measures [159].

Specific clinical areas provide granular, contextual evidence on primary care performance across conditions: palliative care [167,168], musculoskeletal disorders [169], frailty and post-fracture management [170], urology referrals [171], and, in the update, low back pain referrals to neurosurgery, of which 80% (402 of 500) were judged inappropriate against provincial referral criteria with a median consultation delay of 50 days [93], interprofessional chronic-pain services deployed under the 2021–2026 provincial action plan, in which GPs were mainly indirect referrers and nurses acted as the pivot [90], and an integrated youth service in Montreal in which co-located family physicians provided primary care without referral barriers to 719 young people aged 12–25 in one year [72] (retained as an exception to the population rule; Section 2.2). These reports are classified as contextual (Stream B) because they document gaps between recommended and actual care rather than the effect of a named reform; they consistently show that such gaps are largest where interprofessional collaboration or specialist access is required.

### 3.5. Costs and Economic Implications

The economic dimension is comparatively underdeveloped, and the distinction between economic evaluation and resource-utilisation proxies is essential. Only one report contains a formal economic evaluation: the prospective evaluation of NP introduction in long-term care, which reported cost savings [133]. The eConsult scale-up study provides partial costing evidence, reporting cost per eConsult and cost per in-person visit avoided as monitoring metrics without a full economic evaluation [87]. All other “cost” findings are utilisation proxies. Longitudinal analyses reported that team-based care was associated with small reductions in utilisation with potential cost-offset implications, especially among patients with chronic conditions [37,110,148,149,153]; reductions in visits or admissions are not equivalent to demonstrated cost-effectiveness, and no study in the corpus combined them with implementation costs.

Institutional and qualitative reports emphasise that potential efficiency gains are offset by the substantial costs of implementation, including infrastructure, workforce expansion, electronic records, and administrative systems [8,63,64], that the transition toward team-based models requires sustained funding [9,50], and that incentive structures shape economic outcomes [24,25,27,52]; misalignment between fee-for-service incentives and team-based, prevention-oriented objectives is repeatedly identified as a source of inefficiency [24,25,88]. In the update, an implementation project reported that new professional services for unattached patients were financed by pooling physician appointment funds and that below-market salaries hindered physiotherapist recruitment [96], and the expert-committee analysis argued that volume-based indicators limit the assessment of value [100]. Most studies rely on administrative cost proxies rather than evaluations including patient time, informal care, and out-of-pocket costs [172,173,174]; cross-province comparisons indicate that physician supply is a significant driver of provincial health expenditure [173]; and the mandatory universal drug plan, a complementary policy rather than a primary care reform, was associated with improved medication access and modest reductions in income-related inequalities [174]. Overall, the evidence does not support a conclusion on the cost-effectiveness of the reforms.

### 3.6. Equity

Equity evidence is defined here as explicit stratified or distributional analyses; reports that mention vulnerable populations without such analysis are treated as contextual. Across streams, structural inequalities are reported to have persisted despite improvements in overall access [14,32,45,46,128]. Socioeconomic status, geographic location, immigration status, language, gender identity, and health needs continued to shape access, with vulnerable populations facing overlapping barriers [50,68,166,175,176,177,178].

The literature offers a diverse operationalisation of vulnerability: medical criteria, such as the 19 priority diagnostic codes used by the RAMQ to identify medically vulnerable patients eligible for prioritised attachment [12,14,128,129,136]; social indicators such as material and social deprivation, income, education, employment, and language [14,33,146,179]; and multimorbidity, frailty, isolation, low health literacy, and migration status [101,102,162,166,170,176,178]. An individual index of social vulnerability that predicts negative healthcare events provides a more systematic identification tool [179].

Distributional findings (Streams B and C). First, socioeconomic gradients proved resilient to organisational reform: analyses of the evolution of equity in utilisation between 2003 and 2010 found that lower socioeconomic groups continued to benefit less from the reorganisation of primary care and that reform did not substantially reduce pro-rich gradients [45,46]. Centralised waiting lists, despite their inclusive design, were found in a cross-sectional analysis to attach patients from less deprived areas more easily, an effect partially mitigated by prioritisation rules [12,14,128]. The update provides a larger and more recent stratified analysis: in the 2008–2019 cohort, patients attached through the waiting list were older, more often female, more often rural, and more materially and socially deprived, with greater prior healthcare use and poorer health, than patients attached through other routes, whereas younger and healthier urban patients were attached outside the list; age and rurality were more important predictors of attachment route than deprivation [81]. Frequent emergency users with diabetes were characterised by material and social deprivation combined with mental health comorbidity [146]. Among GAP users, younger patients and those in fair or poor health were more likely to report unmet needs [86], and adults aged 66 and over had lower adjusted odds of receiving a physician appointment than younger adults, whereas area deprivation was not clearly associated [98].

Second, specific subpopulations face compounded barriers: recent immigrants report differential utilisation and lower satisfaction [137,176]; patients living in poverty benefit from physicians demonstrating “social competence”, but such practices depend on individual engagement rather than systemic incentives [166,175]; transgender and non-binary people face intersectional barriers with regional disparities [177]; women’s specific needs have been highlighted in policy reports [68,180]; people experiencing homelessness, substance use disorders, and infectious diseases benefit from flexible models such as the SPOT-CCSE clinic [178]; and unattached patients with low incomes, chronic conditions, or limited majority-language proficiency face magnified navigation difficulties, since centralised lists prioritise medical but not social needs [92].

Third, geographic inequities remain entrenched [40,41,42,69,101], Indigenous and remote communities continue to experience structural disadvantages [63,64], and intra-urban variation in provider density and travel time generates inequities within Montreal [39].

Fourth, some reforms may have widened equity gaps. Bill 20 targets and minimum panel requirements are described as creating incentives for adverse selection [8,26,129], and digital appointment systems and telehealth may exacerbate inequities for patients with limited digital literacy, low income, or restricted internet access [51,52,143,144,145]; in the update, the requirement of a health-insurance card and postal address to register on the waiting list, and the digital invitation route of the GAP, are reported to exclude refugees, recent immigrants, and other undocumented groups [85,86]. In the absence of explicit safeguards, reforms are described as having focused on efficiency and overall performance rather than equity [45,46].

Finally, several reports identify conditions under which reforms promoted equity. Waiting lists combined with explicit prioritisation attached more clinically complex patients [12,81,128,136]; FMGs disproportionately served rural and disadvantaged populations relative to non-FMG practices [181] and included patients from lower-income areas [110]; patient navigation, intersectoral interventions, peer support, and tailored programmes narrowed access gaps in single-site evaluations [91,150,152,178]; interprofessional care for unattached patients increased the proportion offered age-appropriate preventive services from 13% to 43% within three months in one clinic [96]. Equity outcomes therefore appear to depend on implementation details, complementary interventions, and explicit equity targets rather than on reform design alone.

### 3.7. Impact on Healthcare Organisation and GP Roles

The transformation of GP roles is one of the most significant and contested outcomes; the evidence is almost entirely qualitative and descriptive (Stream B). The shift toward team-based care expanded the scope of GP responsibilities toward coordination, collaboration, continuity, and the management of chronic disease and multimorbidity [17,75,108,109,115,158], positioning GPs as nodes in integrated networks [48,55,116,182,183]. These changes carry potential for improved delivery but also introduce challenges: administrative workload, organisational complexity, and performance-metric pressure affect satisfaction and engagement [9,26,50]; job satisfaction varies with practice setting, team composition, and patient mix [108,109,184,185]; and tensions between professional autonomy and system-level accountability are recurrent, with some physicians resisting reforms that alter practice patterns and others welcoming collaboration and role expansion [9,10,11,26]. The update adds three illustrations of the persistence of physician-centred organisation at the end of the cycle: in interprofessional chronic-pain services, GP involvement was mainly indirect, as referrer or responding physician, with the nurse acting as pivot [90]; in case-management scale-up, remuneration misaligned with the expected level of involvement limited physician participation [88]; and the expert-committee analysis noted that family physicians remain autonomous professionals in privately owned facilities while allied professionals are employed by the public institution, a structural feature that conditions accountability [100].

Practice setting conditions the nature and quality of care. Comparative cross-sectional studies find that team-based and integrated models support more comprehensive care, broader scope, and higher preventive service delivery [118,121,122,123], and quality indicators favour multidisciplinary practices for patients with chronic conditions [153,184,185]; the relationship between organisational form and quality is mediated by leadership, governance, and team dynamics, so that larger practices are not automatically better [186]. After-hours care and home visits declined or remained limited [130,187]. CLSC physicians, salaried and population-oriented, serve more vulnerable populations than those in private clinics or FMGs [108,109]. Performance assessment of family physicians emerged as a theme, including chart-review reliability and mortality audits [188,189], and Bill 20 reshaped the relationship between physicians, the Ministry, and the FMOQ, generating both compliance and contestation [8,9,10,11]. Physician champions were identified as catalysts of local change in quality-improvement initiatives [99], and the compatibility of residency programmes with advanced access was found to be partial [84]. GP roles evolved during the COVID-19 pandemic with rapid adoption of teleconsultation and remote monitoring [47,48,49,51]; comparative analyses across Belgium, France, Quebec, and Switzerland reveal both convergent adaptations and cross-system variation [47], and the Quebec response highlighted the resilience of the FMG model together with persistent inequities in telehealth access [51,52,144].

### 3.8. Methodological Quality, Evidence Streams, and the Scope Restriction

#### 3.8.1. MMAT Appraisal

Of the 192 included reports, 173 empirical primary studies were appraisable with the MMAT and 19 were not appraisable: 11 policy, institutional, or expert-committee reports [63,64,65,66,67,68,69,70,100,180], one systematic review [53], and seven commentary-style publications [10,11,41,50,80,151,187]. All 173 appraisable reports were appraised (154 from the original search and 19 from the 2026 update). Criterion-level judgements with a justification for every “no” and “can’t tell” are given in Supplementary Material S3, and Table 2 summarises their distribution by MMAT category without computing overall scores. Categories are those assigned at appraisal according to the MMAT design classification and do not coincide with the design taxonomy of Table 3: qualitative descriptive studies that also report quantitative data were appraised as mixed methods, and the four reports containing a randomised component [73,74,75,78] were appraised under the mixed-methods category, with their randomised, non-randomised, descriptive, or qualitative components appraised separately as required by criterion 5.5, so that no report was appraised under the randomised category alone. Of these four reports, only one presents the results of a randomised trial [75]; two are published study protocols describing the design and planned outcomes of randomised evaluations [73,74], and one summarises programme evaluations that included randomised designs [78].

Percentages are computed over the judgements of each category; the row for mixed methods and the total count include only the five mixed-methods criteria. Component-level judgements are not included in the total: the randomised, non-randomised, quantitative descriptive, and qualitative components of the 33 mixed-methods reports (4, 3, 15, and 24 components respectively; 230 judgements: 99 “yes”, 2 “no”, 129 “can’t tell”) are reported in Supplementary Material S3, which also gives the justification of every “no” and “can’t tell” judgement.

Two features of Table 2 matter for the synthesis. First, the reviewers almost never concluded that a study had done something badly: of 865 judgements, 15 (1.7%) were “no” and 542 (62.7%) were “can’t tell”. The criteria that could least often be judged are those demanding methodological detail that health services research based on administrative data and surveys seldom reports: whether the intervention was administered as intended (not assessable for 29 of the 31 non-randomised studies), the representativeness of the sample (not assessable for 56 of the 63 descriptive and 24 of the 31 non-randomised studies), the risk of non-response bias (not assessable for 56 of the 63 descriptive studies and judged low in only two), and, in mixed-methods reports, the handling of divergences between components (33 of 33) and the interpretation of the outputs of integration (32 of 33). Qualitative studies were the most completely reported: the approach was judged appropriate in all 46 and the findings adequately derived from the data in 45, whereas the adequacy of data collection (34 of 46) and the substantiation of the interpretation by the data (35 of 46) could not be judged, mostly because the reports do not describe sampling to saturation or coding procedures, or do not present the data supporting each theme. The 15 “no” judgements concentrate in a few reports and criteria: a high risk of non-response bias in five surveys [86,93,94,95,167], a sample not representative of the target population in three studies [3,155,190], incomplete outcome data and unadjusted confounding in one uncontrolled before-and-after study [83], inappropriate measurement in one survey [93], and an inadequate rationale, integration, and component quality in one quality-improvement report [96]; in addition, the randomised component of the two pragmatic trials [74,75] was judged “no” on the blinding of outcome assessors. The appraisal therefore measures reporting completeness at least as much as methodological quality, and this is itself a substantive finding: the absence of standardised reporting prevents readers and decision-makers from judging how much confidence to place in individual results. Second, the pattern is informative for the synthesis in a specific way. The non-randomised studies (the core of Stream C) are precisely those for which intervention fidelity cannot be assessed, which means that “FMG enrolment” or “attachment” is an exposure of uncertain clinical content in the very analyses that estimate its association with utilisation and processes; the descriptive population and provider surveys (the core of the accessibility evidence) are those for which representativeness and non-response bias cannot be assessed; and the mixed-methods reports, which include most of the multiple-case evaluations of the reforms, seldom document how their components were integrated, so that their qualitative and quantitative findings must be read as juxtaposed rather than triangulated. These limitations are carried into the confidence statements of Table 4.

Beyond the appraisal, the evidence base has structural limitations. Cross-sectional, observational, and descriptive designs dominate [9,20,32,50]; few studies employ designs capable of causal inference [37,38,81,89,110,142,147,148,149,153], and even these are constrained by short follow-up, reliance on administrative indicators, and the difficulty of isolating reform-specific effects from concurrent system changes [53]. Outcome measurement is heterogeneous across patient-reported, provider-reported, and administrative indicators, and across conceptualisations of access, continuity, and equity [32,45,138,139]; the lack of standardised performance frameworks has hampered cumulative learning [63,64,191], although alignment efforts exist, including the QUALICO-PC framework [56,191] and validated patient-experience instruments [160,179]. Patient and public involvement in outcome selection remains a minority practice [76,104,141,158,159,165].

#### 3.8.2. Assessment of Poolability

Meta-analysis was assessed within outcome groups rather than globally. For non-urgent or avoidable emergency department use after FMG enrolment or attachment, four Stream C reports [37,38,81,110] report effect estimates, but they differ in exposure (FMG enrolment, waiting-list attachment), population (diabetic patients, all attached patients), outcome definition (avoidable visits by algorithm, CTAS 4–5 visits, all visits), and estimand (odds ratio, incidence rate ratio, marginal mean difference), and two derive from overlapping RAMQ cohorts; pooling was judged inappropriate and the findings are presented as a directional summary in Table 4. For guideline-concordant processes [110,142,153] the reports derive from the same research programme and dataset and are treated as one study. For post-discharge follow-up [148,149] the two reports are companion analyses of the same cohort. No other outcome group contained more than one comparative estimate.

#### 3.8.3. Reports Not Discussed Thematically

Nine included reports consist of foundational reports, early evaluations, and complementary analyses [78,190,192,193,194,195,196,197] that are not discussed individually in Section 3.2, Section 3.3, Section 3.4, Section 3.5, Section 3.6 and Section 3.7 because their findings are subsumed by later and methodologically stronger reports of the same reforms or research programmes. They are retained in the corpus because they document the pre-reform baseline, provide patient-perception data for periods otherwise unobserved, and corroborate the direction of findings reported.

#### 3.8.4. Impact of the Mental Health Scope Restriction on the Evidence Map

Among the literature, 66 reports were excluded because their primary object was mental health service organisation. Their exclusion affects the evidence map in two identifiable ways. It removes from the accessibility and equity domains a body of evidence on a population, people living with mental disorders, that the retained studies identify as over-represented among frequent emergency users [146], among waiting-list registrants prioritised as medically vulnerable [12,14,127], and among GAP users reporting unmet needs [86]; conclusions on access and equity for this population cannot be drawn from the present review. It does not affect the findings on organisational transformation, structural access, care processes for chronic physical conditions, or costs, for which the excluded reports would have contributed context rather than comparative evidence. The scope restriction is therefore a limitation of coverage rather than a threat to the validity of the retained syntheses.

### 3.9. Summary of the Evidence Base and of Findings

Table 3 reports the distribution of the 192 included reports by evidence stream, study design, main focus, and outcome domain, using the single taxonomy applied in Supplementary Material S5; design and stream are mutually exclusive, whereas main focus and outcome domain follow the primary coding of each report. The vote-count categories of the previously reported table (structural improvement, limited patient-level impact, persistent inequities, heterogeneity of implementation) have been withdrawn because the reports are not independent and the coding rules did not support the precision implied by percentages; the direction and consistency of findings are instead reported by reform mechanism and outcome in Table 4.

Read together with Table 2, the design distribution of Table 3 locates the limitations identified by the appraisal within the evidence base. The 35 comparative evaluative reports of Stream C correspond, with few exceptions, to the studies appraised as quantitative non-randomised or as mixed methods with a randomised or quasi-experimental component, the categories in which intervention fidelity, completeness of outcome data, and control of confounding could least often be judged. The 131 implementation, descriptive, and qualitative reports of Stream B comprise the quantitative descriptive studies, for which representativeness and non-response bias could seldom be judged, and the qualitative and mixed-methods studies, which were the most completely reported on their approach and analysis but not on the integration of their components. The 26 documents of Stream A include the 19 reports that could not be appraised and that were used as contextual sources only (Section 2.7). The confidence attached to each finding in Table 4 therefore reflects, for each reform mechanism and outcome domain, the number of independent studies, their design and evidence stream, and the appraisal profile of their MMAT category.

Three patterns are apparent in Table 4. Confidence is moderate only where policy documents and implementation studies converge on the occurrence of structural change, and where comparative designs converge on a modest, favourable association between team-based care or attachment and utilisation. Confidence is low or very low wherever the outcome is patient-level health, cost-effectiveness, or the effect of legislative and remuneration instruments, either because no comparative design exists or because the available estimates come from a single study or programme. And the evidence added by the 2026 update, while it strengthens the utilisation and equity findings on attachment mechanisms with population-level administrative cohorts, is otherwise descriptive and does not change the confidence level of any cell in the direction of greater certainty about patient-level effects.

## 4. Discussion

### 4.1. Principal Findings and Their Interpretation

This review documents a paradox that the 2026 update sharpens rather than resolves: two decades of primary care reform in Quebec were accompanied by substantial organisational transformation without commensurate, demonstrable improvements in patient-level performance. The restructuring of delivery through FMGs, the formalisation of attachment through centralised waiting lists, and the introduction of accountability mechanisms under Bill 20 are institutional achievements whose occurrence is well documented [6,7,8,9,135]. What the evidence supports about their consequences is more limited and must be stated by evidence stream.

With reasonable confidence, the review supports three statements. Structural change occurred on a large scale, and attachment rates rose over the period, documented consistently by institutional data and registry analyses. Team-based primary care and attachment through the waiting list were associated, in the comparative evaluative studies, with modest reductions in avoidable or non-urgent emergency use and with improvements in guideline-concordant processes [37,38,81,110,142,147,148,149,153]; the magnitude of these associations is small, their distribution across subgroups uneven, and the exposure in each case (enrolment, attachment) is a formal status of uncertain clinical content. Enrolment incentives, evaluated against an interprovincial counterfactual, did not produce a meaningful population-level gain in affiliation [89], which is consistent with the interpretation that attachment mechanisms redistribute a constrained supply rather than expand it.

Suggested primarily by descriptive and qualitative evidence, and therefore held with lower confidence, are the findings that functional integration of teams lagged formal integration, that organisational characteristics rather than attachment status are what patients experience as access, that implementation was highly heterogeneous, and that socioeconomic and geographic gradients persisted. These findings are consistent across many reports, but the reports are largely cross-sectional, often from the same research programmes, and their risk of non-response and selection bias could seldom be assessed.

What remains uncertain is whether the reforms affected health outcomes and costs. The evidence on both is proxy-based, and the absence of evidence of improved population outcomes is not evidence that the reforms produced none: the designs available are largely incapable of detecting such effects, the follow-up periods are short, and the outcomes are shaped by determinants beyond primary care [46,50,110,163,165,166]. What cannot be concluded from this review is anything about the mental health component of primary care, which was outside its scope.

### 4.2. Concurrent Reforms, Secular Trends, and the Limits of Attribution

Any reading of these findings must take account of the concurrent changes against which the reforms unfolded and which the observational designs cannot separate from them. Population ageing and the growth of multimorbidity increased demand throughout the period [102,103,104]; physician retirement cohorts and the regulation of practice location through PREM and AMP altered supply; Bill 10 in 2015 recentralised governance into the CISSS and CIUSSS at the same time as Bill 20 introduced accountability targets; and the COVID-19 pandemic transformed delivery from 2020 onward [47,48,49,50,51,52]. Only the interprovincial and adjusted cohort designs [81,89] and the marginal-structural analyses [110,147] attempt any control for these trends, and even they control for observed characteristics only. Statements in this review that a reform “was associated with” or “was followed by” a change are therefore statements about temporal or adjusted association, and the review makes no causal claim about the effect of any single reform on system performance.

### 4.3. The Structural–Effective Access Distinction as an Organising Framework

The distinction between structural and effective access, developed in the Quebec literature [32,119,138,139], is the most useful framework for reading the evidence and can be expressed as a logic model. Reform inputs and mechanisms (attachment, team composition, scheduling, remuneration, prioritisation, digital access) were expected to produce intermediate structural outputs (enrolment, accredited groups, funded professionals), then effective access (timely appointments, continuity, patient experience), then utilisation changes, clinical outcomes, costs, and equity. The evidence in Table 4 supports the earlier stages of this pathway with moderate confidence and the later stages with low or very low confidence; the point at which confidence drops is the transition from structural outputs to effective access. This is also the point at which the update reports converge: clinic-level access practices, not attachment, predicted patient-reported access [95]; enrolment incentives did not increase affiliation [89]; and GAP users whose request was resolved without a physician appointment reported unmet needs in seven cases out of ten [86]. Mechanisms that maximise attachment, a metric that is easy to track and politically salient, do not by themselves ensure that patients obtain timely, continuous care [17,32,136,140,141], and reform efforts oriented toward effective access would need to combine attachment with same-day capacity, after-hours coverage, and relational continuity [15,16,18,139].

Equity findings fit the same pattern. Waiting lists with prioritisation did reach more deprived, older, rural, and sicker patients [81], consistent with the intended mechanism, yet gradients in utilisation and experience persisted [45,46] and new digital and administrative entry requirements created new exclusions [85,86]. The recommendations on the integration of health, social, and community services for patients with complex needs formulated by a Quebec stakeholder consensus [198] identify the coordination and information-sharing deficits that this review also finds at the root of the gap between registered-clientele panel management and population-based responsibility; and the recent integrative review of collaborative primary care workforce models in Canada, focused on registered-nurse prescribing alongside family physicians and nurse practitioners [199], situates the partial deployment of nurses in Quebec FMGs within a broader pattern in which regulatory authority, training, clinical support tools, role clarity, and remuneration, rather than professional willingness, determine the depth of integration and the safety of task redistribution.

Implementation heterogeneity has both empirical and normative implications. Variation in the depth of FMG implementation, nurse integration, advanced access, and physician engagement [9,15,18,54,82,84,116,128] reflects differences in resources, governance, leadership, and population; some heterogeneity permits local adaptation, but excessive variability undermines equitable access and the spread of innovations [152,200]. The findings also underscore the importance of aligning organisational reforms with incentive structures, workforce policies, and the broader determinants of health [8,25,26,63,65]; where financial incentives shape volume, panel composition, and the patients prioritised [24,25,27], the interaction between reform design, remuneration, and accountability emerges as a central determinant of what reforms deliver.

### 4.4. Policy Developments Since the Reform Cycle

The findings above describe the reform cycle that closed in 2023. The paragraphs that follow are based on legislative and governmental sources rather than on the systematic search, are not the product of a review, and are presented solely as context; they are not evidence of the effectiveness of the arrangements they describe. Three developments are relevant. First, the Act to make the health and social services system more effective (S.Q. 2023, c. 34), adopted in December 2023, created the agency Santé Québec, which assumed the operational governance of the network from December 2024 and altered the administrative structures through which the reforms analysed here were implemented [201]. Second, the GAP, introduced in 2022, has become a routine entry point for unattached patients, and its first evaluations, included in this review, describe its functioning without yet estimating its effects (Section 3.3.4). Third, the Act mainly to establish collective responsibility for improving access to medical services and to ensure the continuity of the provision of those services (Loi visant principalement à instaurer la responsabilité collective quant à l’amélioration de l’accès aux services médicaux et à assurer la continuité de la prestation de ces services), Bill 2, sanctioned on 25 October 2025 (S.Q. 2025, c. 25), gave territorial family medicine departments the mission of affiliating all eligible persons of their territory to practice settings served by family physicians or specialised nurse practitioners, and introduced a remuneration structure in which a share of physician income is conditional on the attainment of collective access and quality targets, with provisions entering into force in stages from 1 January 2026 [202]. The legal text thus establishes collective affiliation alongside, rather than in place of, the existing individual attachment mechanisms. Following an agreement in principle between the government and the Fédération des médecins omnipraticiens du Québec in December 2025, a bill tabled on 4 February 2026 proposed to amend several of these measures and to introduce a blended remuneration model for family physicians in which capitation based on patient enrolment accounts for 50% of remuneration [203]. The adopted provisions and their effective dates should be distinguished from their operational implementation, which was ongoing and contested at the time of writing. The implication for this review is that the attachment-based cycle it synthesises has been succeeded by a phase that again proceeds primarily through organisational and financial instruments; the evidence here constitutes the baseline against which that phase will need to be evaluated, with particular attention to effective access, equity for vulnerable populations, and the role of family physicians within transforming teams [18,52,100,143,144].

### 4.5. Limitations

Five limitations should be acknowledged. First, although the search was rerun in September 2026, eligibility was restricted to evidence on the 2000–2023 reform cycle; the review does not evaluate the post-2023 arrangements and should not be read as a description of current policy. Second, the grey literature was retrieved by structured browsing of institutional repositories whose search functions are limited; the procedure is documented in Supplementary Material S2 but remains less reproducible than the database searches, and some institutional reports may have been missed. Third, the exclusion of reports whose primary object was mental health service organisation, introduced after protocol registration, narrows the scope of the review relative to the full range of primary care activity; its impact on the evidence map is examined in Section 3.8.4, and conclusions on access and equity should not be extended to people living with mental disorders. Fourth, the synthesis is narrative; no meta-analysis was possible within any outcome group (Section 3.8.2), the confidence statements in Table 4 are structured reviewer judgements rather than GRADE assessments. Fifth, the MMAT appraisal measured reporting completeness as much as methodological quality, so that the confidence attached to findings from non-randomised and descriptive studies is limited by what the reports did not say rather than by what they did; and the focus on Quebec constrains generalisability, although comparative studies provide triangulation [55,56,57,204].

## 5. Conclusions

Primary health care reforms implemented in Quebec after 2000 to now represent one of the most sustained efforts to transform the organisation of care within a publicly funded system. Drawing on 191 reports identified up to September 2026, this systematic review characterises what is known about that reform cycle and with what confidence.

Three conclusions can be stated with reasonable confidence. The reforms were accompanied by substantial structural change: FMGs became the dominant organisational form, patient attachment was formalised, and attachment rates rose. Team-based care and waiting-list attachment were associated with modest reductions in avoidable emergency use and improvements in care processes in the comparative evaluations available, with effects that were small, uneven across subgroups, and estimated from exposures of uncertain clinical content. Enrolment incentives did not increase population-level affiliation when evaluated against an interprovincial counterfactual.

Two conclusions are suggested by descriptive and qualitative evidence and are held with lower confidence. Timely and relational access remained difficult even for attached patients, because what patients experience as access depends on clinic-level organisation more than on attachment, and because supply remained constrained. Socioeconomic and geographic inequities persisted, with attachment mechanisms reaching vulnerable groups as intended while new administrative and digital entry requirements created new exclusions.

What remains uncertain is whether the reforms affected health outcomes or costs; the evidence is proxy-based and the designs largely incapable of detecting such effects, so that the absence of demonstrated improvement should not be read as evidence of no effect. What cannot be concluded from this review is anything about the organisation of mental health services in primary care.

For policy, these findings indicate that organisational and financial instruments alone, of the kind that again characterise the post-2023 phase, are unlikely to close the gap between structural and effective access unless combined with same-day capacity, after-hours coverage, relational continuity, explicit equity safeguards, and remuneration aligned with team-based practice. For research, they indicate that the priority is not further description but counterfactual evaluation: natural experiments based on the timing of policies, interprovincial comparisons, linked administrative and patient-reported data with adequate follow-up, and standardised reporting that allows the credibility of findings to be assessed. The evidence accumulated over the 2000–2023 cycle provides the baseline against which the next phase of Quebec primary care policy should be evaluated.

## Figures and Tables

**Table 1:** Glossary of Quebec primary care institutions, access mechanisms, and policy instruments referred to in this review.

Term (French/English)	Definition and Role
GMF/FMG (Groupe de médecine de famille/Family Medicine Group)	Group practice of family physicians, introduced in 2002–2003, to which patients are formally enrolled and which receives public funding for nurses and, from 2015, other allied professionals. The cornerstone organisational model of the reform cycle.
GMF-U/FMG-U (GMF universitaire/University-affiliated FMG)	FMG affiliated with a university department of family medicine, hosting residents; often the site of advanced access and interprofessional innovations.
GMF-R/FMG-R (GMF-Réseau/Network FMG)	FMG with extended opening hours and diagnostic capacity intended to relieve emergency departments (from 2016).
CLSC (Centre local de services communautaires)	Public community health and social service centre, employing salaried physicians and nurses; historically oriented toward population health and vulnerable clienteles.
CISSS/CIUSSS	Integrated (university) health and social service centres created by Bill 10 (2015) through the merger of regional agencies and establishments; since 2024–2025 progressively integrated into the agency Santé Québec.
GACO (Guichet d’accès pour la clientèle orpheline)	Centralised waiting list introduced in 2008 to attach patients without a family physician (“orphan patients”), with prioritisation of medically vulnerable patients.
GAMF (Guichet d’accès à un médecin de famille)	Provincial successor of the GACOs (2016), a single registry for attachment requests.
GAP (Guichet d’accès à la première ligne/Primary Care Access Point)	Introduced in 2022 to orient unattached patients to the appropriate service or professional (physician appointment, nurse, pharmacist, community resource) while they await attachment; the entry point of the post-2023 phase.
Accès adapté/Advanced access	Scheduling model in which providers plan supply against demand and keep a share of appointments open for short-notice needs; diffused in Quebec from 2012.
Loi 20/Bill 20 (2015)	Legislation introducing minimum patient panel requirements and activity targets for family physicians, with financial penalties suspended under an agreement with the FMOQ.
Loi 10/Bill 10 (2015)	Legislation abolishing regional health agencies and creating the CISSS/CIUSSS; a concurrent structural reform.
RAMQ (Régie de l’assurance maladie du Québec)	Provincial health insurance board, which pays physicians (mainly fee-for-service) and administers the public drug plan.
FMOQ (Fédération des médecins omnipraticiens du Québec)	Union of family physicians negotiating remuneration and practice conditions with the Ministry.
DRMG (Département régional de médecine générale)	Regional body of family physicians responsible for the distribution of medical workforce and services within each region.
PREM (Plans régionaux d’effectifs médicaux)	Regional physician workforce plans regulating where new family physicians may establish their practice.
AMP (Activités médicales particulières)	Mandatory participation of family physicians in designated institutional activities (e.g., emergency departments, hospital wards), a longstanding lever of workforce allocation.
EQES (Enquête québécoise sur l’expérience de soins)	Provincial population survey on care experience (2010–2011), a major source of patient-reported access data.
CSBE (Commissaire à la santé et au bien-être)	Independent Health and Welfare Commissioner producing performance appraisals of the health and social services system.
INSPQ/ISQ	Institut national de santé publique du Québec/Institut de la statistique du Québec, producers of population surveys and institutional evaluations.

**Table 2:** Distribution of MMAT 2018 criterion-level judgements among the 173 appraised reports (154 from the original search and 19 from the 2026 update), by MMAT category.

MMAT Category	Reports (*n*)	Judgements (*n*)	“Yes” *n* (%)	“No” *n* (%)	“Can’t Tell” *n* (%)
1. Qualitative	46	230	131 (57.0)	0 (0.0)	99 (43.0)
2. Quantitative randomised (randomised component of four mixed-methods reports [73,74,75,78])	0 (4 components)	20	4 (20.0)	2 (10.0)	14 (70.0)
3. Quantitative non-randomised	31	155	26 (16.8)	3 (1.9)	126 (81.3)
4. Quantitative descriptive	63	315	121 (38.4)	9 (2.9)	185 (58.7)
5. Mixed methods (mixed-methods criteria 5.1–5.5)	33	165	30 (18.2)	3 (1.8)	132 (80.0)
All appraised reports	173	865	308 (35.6)	15 (1.7)	542 (62.7)

**Table 3:** Evidence streams, designs, focus, and outcome domains of the 192 included reports.

Dimension	Category	Reports (*n*)	%
Evidence stream	A. Policy, institutional, review, and commentary documents	26	13.5
	B. Implementation, descriptive, and qualitative studies	131	68.2
	C. Comparative evaluative studies (controlled, quasi-experimental, randomised)	35	18.2
Study design	Qualitative	62	32.3
	Quantitative cross-sectional or descriptive	55	28.6
	Longitudinal, quasi-experimental, or controlled observational	34	17.7
	Mixed methods (including one mixed- methods randomised trial)	27	14.1
	Policy or institutional report	11	5.7
	Systematic or realist review	3	1.6
Main focus	System organisation and organisational models	86	44.8
	FMGs and team-based care	67	34.9
	Care coordination and integration programmes	25	13.0
	Access mechanisms (waiting lists, attachment, GAP)	9	4.7
	Policy instruments (Bill 20, enrolment policies, governance)	5	2.6
Primary outcome domain	Organisation, care processes, and outcomes	101	52.6
	Equity and accessibility	63	32.8
	Accessibility	28	14.6

**Table 4:** Summary of findings by reform mechanism and outcome domain. Reports (*n*)/independent studies or programmes (*n*); designs; predominant direction of finding relative to the reform’s intended objective; consistency across independent studies; reviewer confidence.

Reform Mechanism	Outcome Domain	Reports/Studies (*n*); Designs (Stream)	Direction and Key Estimates	Consistency; Confidence
FMG implementation and team-based care	Organisational transformation (enrolment, team composition)	53 reports/32 studies; Policy reports, cross-sectional, qualitative (A, B)	Favourable: diffusion and formalisation documented; functional integration partial and heterogeneous	High for occurrence of structural change; consistent on partial functional integration Confidence: High for occurrence of structural change; moderate for functional integration.
FMG implementation and team-based care	Timely access and continuity (patient-reported)	18 reports/11 studies; Cross-sectional surveys, multilevel analysis (B)	Mixed: organisational characteristics (nurses, after-hours, advanced access) associated with better reported access; attachment alone not	Consistent across surveys Confidence: Low.
FMG implementation and team-based care	Emergency department use and utilisation	6 reports/3 studies; Quasi-experimental, marginal structural models (C)	Favourable, modest: reductions in avoidable ED visits among diabetic patients [37,38]; small utilisation effects [110,147]	Consistent in direction; heterogeneous by subgroup Confidence: Moderate.
FMG implementation and team-based care	Guideline-concordant processes; post-discharge follow-up	5 reports/2 studies; Longitudinal administrative (C)	Favourable: improved screening and chronic-disease processes [110,142,153]; timelier follow-up [148,149]	Consistent within programme; single programme Confidence: Low.
FMG implementation and team-based care	Health outcomes (clinical, quality of life)	3 reports/2 studies; Mixed-methods RCT [75,76]; cross-sectional [154]	Mixed: feasibility shown, clinical endpoints inconsistent	Insufficient Confidence: Very low.
FMG implementation and team-based care	Costs	2 reports/2 studies; Utilisation proxies (C); no full evaluation	Unclear: utilisation offsets suggested, implementation costs not measured	Insufficient Confidence: Very low.
FMG implementation and team-based care	Equity	4 reports/3 studies; Longitudinal, cross-sectional (B, C)	Mixed: FMGs included lower-income and rural patients [110,181]; gradients in utilisation persisted 2003–2010 [45,46]	Partly consistent Confidence: Low.
Centralised waiting lists and enrolment policies (GACO, GAMF, enrolment incentives)	Structural access (attachment, affiliation)	11 reports/6 studies; Registry analyses (B); difference-in-differences and ITS [89] (C)	Mixed: high throughput of attachments documented; no population-level gain in affiliation from enrolment incentives [89]	Descriptive and comparative evidence diverge Confidence: Moderate for volume of attachments; low for population-level effect on affiliation.
Centralised waiting lists	Emergency department use	1 report/1 study; Adjusted retrospective cohort [81] (C)	Favourable, small: IRR 0.952 (95% CI 0.946–0.960) for non-urgent ED visits after CWL versus non-CWL attachment	Single study Confidence: Low.
Centralised waiting lists	Equity	5 reports/3 studies; Cross-sectional [14,128,129]; cohort [81] (B, C)	Mixed: lists attach more deprived, rural, older, and sicker patients [81] but early evidence of easier attachment from less deprived areas [14]; prioritisation mitigates	Partly consistent over time Confidence: Low.
Advanced access scheduling	Implementation and access practices	8 reports/6 studies; Qualitative, cross-sectional, latent class, realist (B)	Favourable for feasibility; implementation partial and heterogeneous; third-next-available appointment 4–9 days by profile [82]	Consistent on heterogeneity Confidence: Moderate.
Advanced access scheduling	Timely access (patient-reported)	1 report/1 study; Multilevel cross-sectional [95] (B)	Favourable: demand–supply estimation and schedule opening associated with lower difficulty and unmet needs	Single study Confidence: Low.
Bill 20 and accountability instruments	Physician behaviour, organisation	3 reports/2 studies; Policy analysis, qualitative, commentary (A, B)	Unfavourable or uncertain: administrative burden, output focus, adverse-selection risk described; no comparative estimate	Consistent in concern; no effect estimate Confidence: Very low.
Physician remuneration and incentives	Service volume and composition	5 reports/5 studies; ITS [27], econometric [24], mixed-methods [25] (B, C)	Mixed: fee increases associated with volume responses; opting-out associated with policy changes; FFS identified as barrier to team participation [88]	Partly consistent Confidence: Low.
Digital access tools (e-booking, eConsult, telehealth)	Access and specialist referral	8 reports/8 studies; Environmental scan, surveys, case study (B)	Mixed: eConsult avoided in-person specialist visit in >40% of cases (provider report) [94]; e-booking uneven uptake, digital-divide risk [52,143,144]	Consistent on uneven uptake Confidence: Low.
GAP (2022–, transition phase)	Orientation outcomes and unmet needs	3 reports/3 studies; Cross-sectional, multi-case qualitative (B)	Mixed: 64% of requests obtain a physician appointment [98]; unmet needs 17.9% after physician appointment versus 71.8% after advice only [86]	Consistent across reports Confidence: Low.
Targeted programmes (case management, navigation, peer support, chronic disease programmes)	Patient experience, processes, access for vulnerable groups	16 reports/13 studies; Single-site evaluations, qualitative, realist syntheses (B); pre–post [83,96]	Favourable in single-site evaluations; scale-up constrained by remuneration and information systems [88]	Consistent within sites; generalisability uncertain Confidence: Low.
All mechanisms	Population health outcomes (mortality, morbidity)	2 reports/2 studies; Cross-sectional (B)	No measurable association reported [146,154]	Insufficient Confidence: Very low.

## Data Availability

No new data were created or analyzed in this study. Data sharing is not applicable to this article.

## Supplementary Materials

The following supporting information can be downloaded at: https://www.mdpi.com/article/10.3390/healthcare14203373/s1, Supplementary Material S1: completed PRISMA 2020 checklist; Supplementary Material S2: full search strategies for the databases, institutional repositories, and citation searching, with the number of records retrieved from each source; Supplementary Material S3: MMAT 2018 criterion-level appraisal of the 173 appraisable reports, with justification of every “no” and “can’t tell” judgement; Supplementary Material S4: citation-level list of the reports excluded at full text, with primary reason and justification, and crosswalk between the original and revised exclusion categories; Supplementary Material S5: extraction and coding dataset (evidence table), including the report-to-study/programme crosswalk and the coding of evidence stream, reform mechanism, outcome domain, and direction of findings; Supplementary Material S6: rationale for the confidence judgements of Table 4.
