# Supplementary material for: Impact of Primary Health Care Reforms in Quebec’s Health Care System: A Systematic Review
Source: Healthcare (Basel). 2026 Oct 9;14(20):3373. doi: 10.3390/healthcare14203373 (PMC13704467; doi:10.3390/healthcare14203373)
Supplement: Supplementary file 1 [file healthcare-14-03373-s001.zip › healthcare-4520369_R2_S6_Confidence_rationale.pdf]

Supplementary Material S6. Rationale for the confidence statements of Table 4

For each cell of Table 4 (reform mechanism × outcome domain), the confidence attached to the finding is a structured reviewer judgement applying four considerations, as stated in Section 2.7: (1) the study designs available and their capacity for causal inference (evidence stream A, B or C); (2) the MMAT judgements on the criteria most relevant to the outcome (Supplementary Material S3; Table 2); (3) the consistency of the direction of findings across independent studies or programmes (report-to-study crosswalk, Supplementary Material S5); (4) the directness of the outcome measure with respect to the outcome domain. Confidence is expressed on four levels (high, moderate, low, very low). A formal GRADE or GRADE-CERQual assessment was not undertaken (Section 2.7). The confidence levels below are those reported in the confidence column of Table 4.

Default rules applied: high = comparative evidence (C) from at least two independent studies with adequate MMAT profile and consistent direction, or occurrence of a structural change documented by policy and descriptive sources from multiple independent programmes; moderate = several independent studies, consistent direction, but designs unable to support causal inference or relevant MMAT criteria not assessable; low = few studies or a single programme, or relevant MMAT criteria judged "no", or divergent findings, or indirect outcome measures; very low = single study without comparison, no effect estimate, or contradictory findings.

Reform mechanism	Outcome domain	(1) Designs and independent studies	(2) MMAT profile of contributing studies	(3) Consistency	(4) Directness	Confidence	Rationale
FMG implementation and team-based care	Organisational transformation (enrolment, team composition)	Policy reports and registry data (A); cross-sectional surveys and qualitative studies (B); 53 reports / 32 studies	Qualitative criteria well reported (1.1, 1.3, 1.5); representativeness and non-response bias of provider surveys often not assessable (4.2, 4.4)	Consistent across sources on the occurrence of enrolment and team expansion; consistent on partial and heterogeneous functional integration	Direct (structural indicators)	High for the occurrence of structural change; Moderate for the degree of functional integration	Structural change is documented directly by administrative and policy sources from independent programmes; the qualitative and survey evidence on functional integration is consistent but descriptive.

Reform mechanism	Outcome domain	(1) Designs and independent studies	(2) MMAT profile of contributing studies	(3) Consistency	(4) Directness	Confidence	Rationale
FMG implementation and team-based care	Timely access and continuity (patient-reported)	Cross-sectional population surveys and multilevel analyses (B); 18 reports / 11 studies	Non-response bias not assessable in most surveys (4.4 can't tell) and judged high in the largest multilevel survey; representativeness not assessable (4.2)	Consistent across surveys in the association between organisational characteristics and reported access; attachment alone not associated	Direct (patient-reported access)	Low	Associations are consistent but cross-sectional, self-reported and exposed to non-response bias that cannot be excluded.
FMG implementation and team-based care	Emergency department use and utilisation	Quasi-experimental designs and marginal structural models on administrative data (C); 6 reports / 3 independent studies	Intervention administered as intended not assessable (3.5); confounding addressed by design; complete outcome data in administrative cohorts	Consistent in direction across the three studies; heterogeneous by subgroup and modest in size	Direct (administrative utilisation)	Moderate	Designs capable of inference, consistent direction from independent studies; downgraded for non-assessable fidelity of the exposure (content of FMG enrolment) and heterogeneity of effect.

**Reform mechanism**	**Outcome domain**	**(1) Designs and independent studies**	**(2) MMAT profile of contributing studies**	**(3) Consistency**	**(4) Directness**	**Confidence**	**Rationale**
FMG implementation and team-based care	Guideline-concordant processes; post-discharge follow-up	Longitudinal administrative analyses (C); 5 reports from 2 studies within a single research programme	3.5 not assessable; outcome data complete; confounding partly addressed	Consistent within the programme; no independent replication	Direct (process indicators)	Low	Favourable and consistent, but a single programme and administrative process measures; independent replication absent.
FMG implementation and team-based care	Health outcomes (clinical, quality of life)	Mixed-methods pragmatic RCT and its process evaluation; one cross-sectional study; 3 reports / 2 studies	Randomised component: blinding of outcome assessors judged "no" (2.4); other RCT criteria not assessable; integration criteria (5.3, 5.4) not assessable	Insufficient: feasibility shown, clinical endpoints inconsistent	Direct but limited to one intervention	Very low	Single trial with inconsistent endpoints and non-assessable trial criteria; no other comparative evidence.
FMG implementation and team-based care	Costs	Utilisation proxies from comparative studies (C); no full economic evaluation; 2 reports / 2 studies	As for utilisation; costs not measured directly	Insufficient	Indirect (utilisation offsets, no implementation costs)	Very low	No economic evaluation; utilisation reductions are not evidence of cost-effectiveness.

Reform mechanism	Outcome domain	(1) Designs and independent studies	(2) MMAT profile of contributing studies	(3) Consistency	(4) Directness	Confidence	Rationale
FMG implementation and team-based care	Equity	Longitudinal and cross-sectional analyses (B, C); 4 reports / 3 studies	Representativeness of population surveys not assessable; administrative analyses with adjustment	Partly consistent: inclusion of lower-income and rural patients documented; utilisation gradients persisted 2003–2010	Direct (stratified analyses)	Low	Findings are stratified but few, partly divergent, and based on older data.
Centralised waiting lists and enrolment policies	Structural access (attachment, affiliation)	Registry analyses (B) and one difference-in-differences / ITS study (C); 11 reports / 6 studies	Registry descriptive data (4.4 not applicable); the comparative study meets 3.1–3.4, 3.5 not assessable	Descriptive and comparative evidence diverge: high throughput of attachments versus no population-level gain in affiliation from incentives	Direct for throughput; direct for affiliation rate	Moderate for the volume of attachments achieved; Low for a population-level effect on affiliation	Throughput is documented by registry data from independent sources; the only comparative estimate finds no population-level effect and is not replicated.
Centralised waiting lists	Emergency department use	Adjusted retrospective cohort (C); 1 report / 1 study	3.1–3.4 met; 3.5 not assessable	Single study	Direct	Low	Small favourable effect (IRR 0.952) from one adjusted cohort without replication.

Reform mechanism	Outcome domain	(1) Designs and independent studies	(2) MMAT profile of contributing studies	(3) Consistency	(4) Directness	Confidence	Rationale
Centralised waiting lists	Equity	Cross-sectional analyses (B) and one cohort (C); 5 reports / 3 studies	Early cross-sectional studies: representativeness and non-response not assessable; cohort adjusted	Partly consistent over time: lists attach more deprived and sicker patients, but early evidence of easier attachment from less deprived areas	Direct (deprivation-stratified)	Low	Direction changed over time with prioritisation; few independent studies.
Advanced access scheduling	Implementation and access practices	Qualitative, cross-sectional, latent class and realist studies (B); 8 reports / 6 studies	Qualitative criteria largely met; 1.2 and 1.4 not assessable in some reports; mixed-methods integration criteria not assessable	Consistent on partial and heterogeneous implementation across reports and sites	Direct for implementation; indirect for access (third-next-available appointment by profile)	Moderate for feasibility and heterogeneity of implementation	Consistent, well-reported qualitative and descriptive evidence from several sites; no comparative estimate of effect on access.
Advanced access scheduling	Timely access (patient-reported)	Multilevel cross-sectional survey (B); 1 report / 1 study	Non-response bias judged “no” (4.4); representativeness not assessable	Single study	Direct (patient-reported)	Low	Large single survey with high risk of non-response bias; associations not replicated.

Reform mechanism	Outcome domain	(1) Designs and independent studies	(2) MMAT profile of contributing studies	(3) Consistency	(4) Directness	Confidence	Rationale
Bill 20 and accountability instruments	Physician behaviour, organisation	Policy analysis, qualitative studies, commentaries (A, B); 3 reports / 2 studies	Not appraisable sources predominate; qualitative studies well reported	Consistent in the concerns described; no effect estimate	Indirect (perceived and anticipated effects)	Very low	No comparative estimate of effect; consistency concerns perceptions rather than measured behaviour.
Physician remuneration and incentives	Service volume and composition	ITS, econometric analysis, mixed-methods study (B, C); 5 reports / 5 studies	ITS and econometric studies: 3.5 not assessable, confounding addressed; mixed-methods integration not assessable	Partly consistent: volume responses to fee changes; FFS identified as barrier to team participation	Direct (billing volumes)	Low	Few heterogeneous studies on different instruments; consistent only at the level of the general mechanism.
Digital access tools (e-booking, eConsult, telehealth)	Access and specialist referral	Environmental scan, provider surveys, case study (B); 8 reports / 8 studies	Provider surveys: representativeness and non-response not assessable; outcomes provider-reported	Consistent on uneven uptake and digital-divide risk	Indirect (provider-reported avoided visits)	Low	Descriptive and provider-reported evidence without comparison group.
GAP (2022–, transition phase)	Orientation outcomes and unmet needs	Cross-sectional (administrative and patient survey) and	Patient survey: non-response bias judged “no”; administrative	Consistent across reports of the same programme	Direct (orientation outcomes;	Low	Recent, single-programme, cross-sectional evidence with

**Reform mechanism**	**Outcome domain**	**(1) Designs and independent studies**	**(2) MMAT profile of contributing studies**	**(3) Consistency**	**(4) Directness**	**Confidence**	**Rationale**
		multi-case qualitative studies (B); 3 reports / 3 studies within one research programme	study: low missing data; qualitative study well reported		patient-reported unmet needs)		acknowledged selection bias; no comparison with the pre-GAP situation.
Targeted programmes (case management, navigation, peer support, chronic disease programmes)	Patient experience, processes, access for vulnerable groups	Single-site evaluations, qualitative studies, realist syntheses (B); uncontrolled pre–post studies; 16 reports / 13 studies	Pre–post studies: incomplete outcome data and unadjusted confounding judged "no" in one; qualitative criteria largely met	Consistent within sites; generalisability uncertain	Direct for experience and processes; indirect for access	Low	Favourable single-site evaluations without control groups; scale-up constraints consistently reported.
All mechanisms	Population health outcomes (mortality, morbidity)	Cross-sectional (B); 2 reports / 2 studies	Descriptive; representativeness not assessable	Insufficient	Indirect	Very low	No comparative evidence; absence of a measurable association is not evidence of absence of effect (Section 4.1).
