# Supplementary material for: Impact of Primary Health Care Reforms in Quebec’s Health Care System: A Systematic Review
Source: Healthcare (Basel). 2026 Oct 9;14(20):3373. doi: 10.3390/healthcare14203373 (PMC13704467; doi:10.3390/healthcare14203373)
Supplement: Supplementary file 1 [file healthcare-14-03373-s001.zip › healthcare-4520369_R2_S2_Search_strategies.pdf]

Supplementary Material S2. Search strategies

Records retrieved. The “Total number of results” reported for each database is the number of records retrieved by the original search (7 November 2023) and the update (3 September 2026) combined (PubMed 325, Embase 647, Web of Science 554, CINAHL 443; total 1,969). Test runs of the same strategies, performed before the original search to estimate the screening workload, were also imported into Covidence and are counted with the original search in the PRISMA flow diagram (3,366 database records in total; manuscript, Section 3.1 and Figure 1). The update was limited to records added to each database after the original search (line 5 of each strategy).

PubMed

Date of the initial search: 07-11-2023

Date of the updated search: 03-09-2026

Database limit: No database limit has been applied.

#	Search strategy	Results
1	"Primary Health Care"[Mesh:NoExp] OR Primary Care[TIAB] OR Primary Healthcare[TIAB] OR Primary Health Care[TIAB] OR "Physicians, Family"[Mesh] OR Family Physician*[TIAB] OR Family Practi*[TIAB] OR "General Practitioners"[Mesh] OR "General Practice"[Mesh] OR General Practi*[TIAB]	
2	"Health Services Needs and Demand"[Mesh] OR "Health Services Accessibility"[Mesh:NoExp] OR "Delivery of Health Care"[Mesh:NoExp] OR "Health Care Reform"[Mesh] OR "Health Policy"[Mesh:NoExp] OR "Appointments and Schedules"[Mesh:NoExp] OR "Mass Screening/organization and administration"[Mesh:NoExp] OR Outcome and Process Assessment, Health Care[Mesh] OR Quality Indicators, Health Care[Mesh:NoExp] OR Waiting Lists[Mesh] OR Health Polic*[TIAB] OR Healthcare Polic*[TIAB] OR National Polic*[TIAB] OR Healthcare Delivery[TIAB] OR delivery of care[TIAB] OR Health access*[TIAB] OR Healthcare access*[TIAB] OR Health Care Reform*[TIAB] OR Health demand[TIAB] OR care demand[TIAB]	
3	"Quebec"[Mesh] OR Quebec[TIAB]	
4	#1 AND #2 AND #3	292
5	#4 AND (2023/11/08:2026/09/03[edat] OR 2023/11/08:2026/09/03[crdt])	33
	**Total number of results**	325

Embase (Embase.com)

Date of the initial search: 07-11-2023

Date of the updated search: 03-09-2026

Database limit: for the update, only articles were considered.

#	**Search strategy**	**Results**
1	'primary health care'/exp OR 'general practitioner'/de OR 'general practice'/de OR (Primary NEAR/2 (Care OR Healthcare OR "Health Care")):ti,ab,kw OR ((Family OR General) NEAR/2 (Practi* OR Physician*)):ti,ab,kw	
2	'health care need'/de OR 'health care access'/de OR 'health care delivery'/de OR 'health care policy'/de OR 'health care quality'/de OR ((Health OR Healthcare OR National) NEAR/2 Polic*):ti,ab,kw OR ((Healthcare OR Health OR care) NEAR/2 (Delivery OR access*)) OR ((Healthcare OR Health OR care) NEAR/2 (demand OR Reform*)):ti,ab,kw OR ((Healthcare OR Health OR care) NEAR/2 quality):ti,ab,kw OR ((care OR health OR healthcare) NEAR/1 (management OR organisation OR reorganisation OR planning OR expenditure OR cost* OR outcome* OR system OR indicators OR performance OR services OR demand)):ti,ab,kw	
3	'Quebec'/de OR Quebec:ti,ab,kw	
4	#1 AND #2 AND #3	535
5	#4 AND [08-11-2023]/sd -article results database limit	112
	**Total number of results**	647

Web of Science

Date of the initial search: 07-11-2023

Date of the updated search: 03-09-2026

Database limit: No database limit has been applied.

#	Search strategy	Results
1	TS=(Primary NEAR/2 (Care OR Healthcare OR "Health Care")) OR TS=((Family OR General) NEAR/2 (Practi* OR Physician*))	
2	TS=((Health OR Healthcare OR National) NEAR/2 Polic*) OR TS=((Healthcare OR Health OR care) NEAR/2 (Delivery OR access*)) OR TS=((Healthcare OR Health OR care) NEAR/2 (demand OR Reform*)) OR TS=((Healthcare OR Health OR care) NEAR/2 quality) OR TS=((care OR health OR healthcare) NEAR/1 (management OR organisation OR reorganisation OR planning OR expenditure<br><br>OR cost* OR outcome* OR system OR indicators OR performance OR services OR demand))	
3	TS=(Quebec)	
4	#1 AND #2 AND #3	463
5	#4 AND LD=(2023-11-08/2026-09-03)	91
	**Total number of results**	554

CINAHL (EBSCO)

Date of the initial search: 07-11-2023

Date of the updated search: 03-09-2026

Database limit: No database limit has been applied.

#	Search strategy	Results
1	MH "Primary Health Care" OR MH "Physicians, Family" OR MH "Family Practice" OR TI (Primary N2 (Care OR Healthcare OR "Health Care")) OR AB (Primary N2 (Care OR Healthcare OR "Health Care")) OR TI ((Family OR General) N2 (Practi* OR Physician*)) OR AB ((Family OR General) N2 (Practi* OR Physician*)	
2	MH "Health Services Needs and Demand" OR MH "Health Services Accessibility" OR MH "Health Care Delivery" OR MH "Health Care Reform" OR MH "Health Policy" OR MH "Appointments and Schedules" OR MH "Clinical Indicators" OR TI ((Health OR Healthcare OR National) N2 Polic*)<br><br>OR AB ((Health OR Healthcare OR National) N2 Polic*) OR TI ((Healthcare OR Health OR care) N2 (Delivery OR access*)) OR AB ((Healthcare OR Health OR care) N2 (Delivery OR access*)) OR TI ((Healthcare OR Health OR care) N2 (demand OR Reform*)) OR AB ((Healthcare OR Health OR care) N2 (demand OR Reform*)) OR TI ((Healthcare OR Health OR care) N2 quality) OR AB ((Healthcare OR Health OR care) N2 quality) OR TI ((care OR health OR healthcare) N1 (management OR organisation OR reorganisation OR planning OR expenditure OR cost* OR outcome* OR system OR indicators OR performance OR services OR demand)) OR AB ((care OR health OR healthcare) N1 (management OR organisation OR reorganisation OR planning OR expenditure OR cost* OR outcome* OR system OR indicators OR performance OR services OR demand))	
3	MH Quebec OR TI Quebec OR AB Quebec	
4	S1 AND S2 AND S3	395
5	S4 AND EM 20231108-20260903	48
	**Total number of results**	443

Institutional repositories and other methods

Sources. Online repositories and publication catalogues of the following institutions: Commissaire à la santé et au bien-être; Institut national de santé publique du Québec (INSPQ); Institut de la statistique du Québec; Ministère de la Santé et des Services sociaux (publications catalogue); Institut national d'excellence en santé et en services sociaux (INESSS); Fédération des médecins omnipraticiens du Québec; Conseil du statut de la femme; and the Santécom catalogue of the libraries of the Quebec health and social services network.

Procedure. Each source was browsed in a structured way at the time of the original search and again at the update, using terms corresponding to the concepts of the database strategies, in French and English (primary care / première ligne; family medicine, family physician, Family Medicine Groups / médecine familiale, médecin de famille, groupes de médecine de famille; access / accessibilité; reform, organisation of services / réforme, organisation des services; Quebec / Québec), and limited to documents published from January 2000 onwards. Documents reporting empirical data on Quebec primary care were exported in bibliographic format.

Records retrieved. The repository records were imported into Covidence in a single batch (85 records; 74 after deduplication against the database records) and were screened together with the database records, with the same eligibility criteria and procedure; 25 of the 192 included reports come from these sources. The check at the update identified no additional record not already in the review.

Backward citation searching. The reference lists of all included reports were checked at the original search and at the update; this procedure identified no report that had not already been retrieved by the database or repository searches.
