# Supplementary material for: Impact of Primary Health Care Reforms in Quebec’s Health Care System: A Systematic Review
Source: Healthcare (Basel). 2026 Oct 9;14(20):3373. doi: 10.3390/healthcare14203373 (PMC13704467; doi:10.3390/healthcare14203373)
Supplement: Supplementary file 1 [file healthcare-14-03373-s001.zip › healthcare-4520369_R2_S1_PRISMA_checklist.pdf]

Supplementary Material S1. PRISMA 2020 checklist

Completed PRISMA 2020 checklist (Page et al., BMJ 2021;372:n71) for: Impact of Primary Health Care Reforms in Quebec's Health Care System: A Systematic Review. Locations refer to section numbers of the manuscript; page numbers are to be added on the typeset proof.

Section	Topic	Item	Checklist item	Location where item is reported
TITLE	Title	1	Identify the report as a systematic review.	Title page
ABSTRACT	Abstract	2	See the PRISMA 2020 for Abstracts checklist.	Abstract (structured: background, objective, methods, results, conclusions)
INTRODUCTION	Rationale	3	Describe the rationale for the review in the context of existing knowledge.	Section 1 (Introduction), paragraphs 1–4
	Objectives	4	Provide an explicit statement of the objective(s) or question(s) the review addresses.	Section 1, final paragraph
METHODS	Eligibility criteria	5	Specify the inclusion and exclusion criteria for the review and how studies were grouped for the syntheses.	Section 2.2; grouping by evidence stream and reform mechanism: Section 2.7
	Information sources	6	Specify all databases, registers, websites, organisations, reference lists and other sources searched or consulted to identify studies. Specify the date when each source was last searched or consulted.	Section 2.3; Supplementary Material S2

Section	Topic	Item	Checklist item	Location where item is reported
	Search strategy	7	Present the full search strategies for all databases, registers and websites, including any filters and limits used.	Supplementary Material S2 (PRISMA-S)
	Selection process	8	Specify the methods used to decide whether a study met the inclusion criteria of the review, including how many reviewers screened each record and each report retrieved, whether they worked independently, and if applicable, details of automation tools used in the process.	Section 2.4
	Data collection process	9	Specify the methods used to collect data from reports, including how many reviewers collected data from each report, whether they worked independently, any processes for obtaining or confirming data from study investigators, and if applicable, details of automation tools used in the process.	Section 2.5
	Data items	10a	List and define all outcomes for which data were sought. Specify whether all results that were compatible with each outcome domain in each study were sought, and if not,	Section 2.5; outcome domains defined in Section 2.2 and Section 2.7

Section	Topic	Item	Checklist item	Location where item is reported
			the methods used to decide which results to collect.	
		10b	List and define all other variables for which data were sought. Describe any assumptions made about any missing or unclear information.	Section 2.5; Supplementary Material S5
	Study risk of bias assessment	11	Specify the methods used to assess risk of bias in the included studies, including details of the tool(s) used, how many reviewers assessed each study and whether they worked independently, and if applicable, details of automation tools used in the process.	Section 2.6 (MMAT 2018); Supplementary Material S3
	Effect measures	12	Specify for each outcome the effect measure(s) (e.g. risk ratio, mean difference) used in the synthesis or presentation of results.	Section 2.7 (SWiM items; measures reported as in the primary reports)
	Synthesis methods	13a	Describe the processes used to decide which studies were eligible for each synthesis.	Section 2.7 (evidence streams; reform mechanism × outcome domain)
		13b	Describe any methods required to prepare the data for presentation or synthesis, such as handling of missing	Section 2.7; Supplementary Material S5

Section	Topic	Item	Checklist item	Location where item is reported
			summary statistics, or data conversions.	
		13c	Describe any methods used to tabulate or visually display results of individual studies and syntheses.	Section 2.7; Tables 3 and 4; Figure 1
		13d	Describe any methods used to synthesize results and provide a rationale for the choice(s). If meta-analysis was performed, describe the model(s), method(s) to identify the presence and extent of statistical heterogeneity, and software package(s) used.	Section 2.7 (narrative synthesis; SWiM; Popay et al.); assessment of poolability: Section 3.8.2
		13e	Describe any methods used to explore possible causes of heterogeneity among study results (e.g. subgroup analysis, meta-regression).	Section 2.7 (heterogeneity examined by design, period and population within outcome groups)
		13f	Describe any sensitivity analyses conducted to assess robustness of the synthesized results.	Not applicable (no quantitative pooling); robustness examined through the report-to-study crosswalk (Section 2.5) and the MMAT filter (Section 2.7)

Section	Topic	Item	Checklist item	Location where item is reported
	Reporting bias assessment	14	Describe any methods used to assess risk of bias due to missing results in a synthesis (arising from reporting biases).	Not formally assessed; discussed in Section 4.5 (Limitations)
	Certainty assessment	15	Describe any methods used to assess certainty (or confidence) in the body of evidence for an outcome.	Section 2.7 (structured reviewer confidence statements); Supplementary Material S6
RESULTS	Study selection	16a	Describe the results of the search and selection process, from the number of records identified in the search to the number of studies included in the review, ideally using a flow diagram.	Section 3.1; Figure 1
		16b	Cite studies that might appear to meet the inclusion criteria, but which were excluded, and explain why they were excluded.	Section 3.1; Supplementary Material S4
	Study characteristics	17	Cite each included study and present its characteristics.	Section 3.1; Table 3; Supplementary Material S5
	Risk of bias in studies	18	Present assessments of risk of bias for each included study.	Section 3.8.1; Table 2; Supplementary Material S3

Section	Topic	Item	Checklist item	Location where item is reported
	Results of individual studies	19	For all outcomes, present, for each study: (a) summary statistics for each group (where appropriate) and (b) an effect estimate and its precision (e.g. confidence/credible interval), ideally using structured tables or plots.	Sections 3.2–3.7 (key estimates in text); Table 4; Supplementary Material S5 (extraction dataset)
	Results of syntheses	20a	For each synthesis, briefly summarise the characteristics and risk of bias among contributing studies.	Table 4; Section 3.9
		20b	Present results of all statistical syntheses conducted. If meta-analysis was done, present for each the summary estimate and its precision and measures of statistical heterogeneity. If comparing groups, describe the direction of the effect.	Sections 3.2–3.7; Table 4 (direction and key estimates)
		20c	Present results of all investigations of possible causes of heterogeneity among study results.	Section 3.8.2; Table 4 (consistency column)
		20d	Present results of all sensitivity analyses conducted to assess the robustness of the synthesized results.	Not applicable
	Reporting biases	21	Present assessments of risk of bias due to missing results	Not assessed (Section 4.5)

Section	Topic	Item	Checklist item	Location where item is reported
			(arising from reporting biases) for each synthesis assessed.	
	Certainty of evidence	22	Present assessments of certainty (or confidence) in the body of evidence for each outcome assessed.	Table 4 (confidence column); Supplementary Material S6
DISCUSSION	Discussion	23a	Provide a general interpretation of the results in the context of other evidence.	Sections 4.1–4.3
		23b	Discuss any limitations of the evidence included in the review.	Sections 3.8, 4.5
		23c	Discuss any limitations of the review processes used.	Section 4.5
		23d	Discuss implications of the results for practice, policy, and future research.	Sections 4.3, 4.4; Conclusions
OTHER INFORMATION	Registration and protocol	24a	Provide registration information for the review, including register name and registration number, or state that the review was not registered.	Section 2.1 (PROSPERO CRD42023421145)
		24b	Indicate where the review protocol can be accessed, or state that a protocol was not prepared.	Section 2.1 (published protocol [60])

Section	Topic	Item	Checklist item	Location where item is reported
		24c	Describe and explain any amendments to information provided at registration or in the protocol.	Sections 2.2 and 2.8 (mental health exclusion; search update)
	Support	25	Describe sources of financial or non-financial support for the review, and the role of the funders or sponsors in the review.	Funding statement
	Competing interests	26	Declare any competing interests of review authors.	Conflicts of Interest statement
	Availability of data, code and other materials	27	Report which of the following are publicly available and where they can be found: template data collection forms; data extracted from included studies; data used for all analyses; analytic code; any other materials used in the review.	Data Availability Statement; Supplementary Materials S2–S6

PRISMA 2020 for Abstracts checklist

Topic	Item	Checklist item	Reported
Title	1	Identify the report as a systematic review.	Title
Objectives	2	Provide an explicit statement of the main objective(s) or question(s) the review addresses.	Abstract – Objective
Eligibility criteria	3	Specify the inclusion and exclusion criteria for the review.	Abstract – Methods

Topic	Item	Checklist item	Reported
Information sources	4	Specify the information sources (e.g. databases, registers) used to identify studies and the date when each was last searched.	Abstract – Methods
Risk of bias	5	Specify the methods used to assess risk of bias in the included studies.	Abstract – Methods (MMAT)
Synthesis of results	6	Specify the methods used to present and synthesise results.	Abstract – Methods (narrative synthesis, SWiM)
Included studies	7	Give the total number of included studies and participants and summarise relevant characteristics of studies.	Abstract – Results (192 reports; streams)
Synthesis of results	8	Present results for main outcomes, preferably indicating the number of included studies and participants for each. If meta-analysis was done, report the summary estimate and confidence/credible interval.	Abstract – Results
Limitations of evidence	9	Provide a brief summary of the limitations of the evidence included in the review.	Abstract – Results/Conclusions
Interpretation	10	Provide a general interpretation of the results and important implications.	Abstract – Conclusions
Funding	11	Specify the primary source of funding for the review.	Funding statement (not in abstract)
Registration	12	Provide the register name and registration number.	Abstract – Methods (PROSPERO CRD42023421145)
